# Microbiome and Blood Analyte Differences Point to Community and Metabolic Signatures in Lean and Obese Horses

**DOI:** 10.3389/fvets.2018.00225

**Published:** 2018-09-20

**Authors:** Amy S. Biddle, Jean-Francois Tomb, Zirui Fan

**Affiliations:** ^1^Department of Animal and Food Science, University of Delaware, Newark, DE, United States; ^2^Department of Computer and Information Sciences, University of Delaware, Newark, DE, United States

**Keywords:** equine gut microbiome, obesity, 16S rRNA, network analysis, insulin, leptin, triglycerides, glucose

## Abstract

Due to modern management practices and the availability of energy dense feeds, obesity is a serious and increasingly common health problem for horses. Equine obesity is linked to insulin resistance and exacerbation of inflammatory issues such as osteoarthritis and laminitis. While the gut microbiome is thought to play a part in metabolic status in horses, bacterial communities associated with obesity have yet to be described. Here we report differences in metabolic factors in the blood of obese, normal and lean horses correlated with differences in gut microbiome composition. We report that obese horses had higher levels of leptin, triglycerides, glucose, and cortisol in their blood, and more diverse gut microbiome communities with higher relative abundance of Firmicutes, and lower numbers of Bacteroidetes and Actinobacteria. Network analyses of correlations between body condition, blood analytes, and microbial composition at the genus level revealed a more nuanced picture of microbe-host interactions, pointing to specific bacterial species and assemblages that may be signatures of obesity and leanness in the horse gut. In particular, bacteria groups positively associated with two blood analytes and obesity included *Butyrivibrio* spp., Prevotellaceae, *Blautia* spp., two members of Erysipelotrichaceae, and a Lachnospiraceae taxa. These results are an important first step in unraveling the metabolic differences between obese and lean horse gut communities, and designing targeted strategies for microbial intervention.

## Introduction

As hindgut fermenting, obligate herbivores, horses rely on the gut microbiome to access nutrients and energy from dietary complex carbohydrates. Short chain fatty acids produced by microbial metabolism have been estimated to provide as much as 42% of equine energy needs (1, 2). Surveys of the equine gut microbiome using 16S rDNA sequencing have revealed communities dominated by Firmicutes, and Bacteroidetes (comprising 75% or greater relative abundance), with less abundant Proteobacteria, Verrucomicrobia, Spirochaetes, Actinobacteria, and Fibrobacteres (3–7). As with other animal and human studies, the horse gut microbiome is sensitive to diet, specifically consumption of starch (8–11), fiber (9, 12–14), and high fat (9, 15), or following a rapid change in diet (14, 16). Both age (17) and exercise (18, 19) have also been shown to impact the composition of the equine gut microbiome.

Paralleling human health trends, equine obesity is a growing problem for horse owners, managers, and veterinarians despite greater awareness of body condition assessment, and the availability of specialized feeds for weight management (20–23). A recent study of 300 horses in Virginia using a standardized 1–9 scale to estimate body condition score (BCS) (24), found as many as 51% to be over-conditioned or obese (22). Indications suggest that this estimate of obesity is not an isolated trend (20, 23, 25).

A primary component of Equine Metabolic Syndrome (EMS), obesity contributes to insulin resistance (26–30), predisposes horses to laminitis (30–32), exacerbates heat intolerance(33), reduces performance (34, 35), and increases joint stress (36, 37). A breed effect in the incidence of EMS indicators has been demonstrated, with higher prevalence in ponies, Standardbreds, Andalusians (38), and Rocky Mountain Horses (39), and lower rates in Thoroughbreds, Quarter Horses, and mixed breeds (39).

While human and mouse studies comparing the gut microbiomes of lean and obese individuals have shown a higher Firmicutes: Bacteroidetes ratio correlated with obesity (40–42), comparisons of fewer than 10 obese and lean horses have observed no difference in the ratio of these groups (12, 43). One comparative EMS study of 20 horses found specific genera associated with obesity, including: *Clostridium* cluster XI, *Lactobacillus, Cellulosilyticum, Elusimicrobium*, and members of the phyla Verrucomicrobia, while *Fibrobacter, Ruminococcus, Saccharofermentans, Anarovorax*, and members of Lachnospiraceae and Rhodospirillaceae families were correlated with normal controls (12).

Several metabolic markers in blood have been shown to be correlated with high BCS in horses, namely higher levels of resting insulin, glucose, leptin, adiponectins, and triglycerides (39, 44–46). Higher leptin levels have been shown to be especially pronounced in horses fed diets rich in cereals or fat (38), but no response was seen in obese horses fed varying levels of non-structural carbohydrates in hay (47). Additionally, horses with higher levels of leptin showed elevated insulin (44, 46) and cortisol (especially mares) (44).

While levels of obesity associated blood analytes have been described in horses, studies to identify differences in the gut microbiomes of obese and lean horses have been few and limited to a small number of horses. The purpose of the present study is to correlate blood metabolites related to EMS (insulin, glucose, triglycerides, leptin, ACTH, and cortisol) with gut microbiome differences in a set of 78 horses: lean (*n* = 24), normal (*n* = 17), and obese (*n* = 37).

## Materials and methods

### Fecal sample collection

Fecal samples were collected manually midrectum from horses before breakfast, and stored in ice for no more than 2 h prior to storage at −80°C. Sampling was done in the January–April of 2015 or 2016, before horses had access to fresh, spring grass. Pasture-fed horses were of various breeds, aged 2–20 years, from three university herds: (University of Massachusetts, Amherst, MA, University of Illinois, Champaign-Urbana, IL, or Virginia-Maryland Regional College of Veterinary Medicine, Blacksburg, VA) or private horse owners from five different farms. To minimize variation due to location or owner, no facility contained fewer than three horses. Horses that had received antibiotic or anthelmintic medication within 60 days of collection were removed from the study. Metadata collected for each horse included: breed, gender, diet, housing type, and age. Diet was divided into three categories depending on primary feed with no distinction made for quantity or quality. Diet categories included: Pasture (P), Hay (H), Hay, and concentrate (HC). Age was divided into two categories: 10 years or less (Age_Y, *n* = 29), and over 10 years (Age_M, *n* = 49). BCS (1-9) was determined by the average of at least three observers using the Hennecke scale (24). Horses with divergent BCS across body regions were not sampled due to the possibility of metabolic issues. Classifications of obese, normal, or lean were assigned to each horse based on score: 7 or higher, between 6 and 7, and 5.5 and less, respectively. The demographics of horses participating in this study are summarized in Table 1.

**Table 1:** Demographics of horses included in this study (for complete horse list, see Table S1).

**Farm**	**Horses**	**Gender**			**Age**		**BCS category**			**Feed**		
	**Total**	**Stallion**	**Mare**	**Gelding**	**Y**	**M**	**Lean**	**Normal**	**Obese**	**P**	**H**	**HC**
IU	6	3	3	0	4	2	1	0	5	6	0	0
UM	14	0	4	10	5	9	0	0	14	0	14	0
VM	25	0	17	8	4	21	11	0	14	25	0	0
PO-DE	18	0	14	4	15	3	10	7	1	0	0	18
PO-NH	15	0	5	10	2	13	2	10	3	0	0	15
Total	78	3	43	32	30	48	24	17	37	31	14	33

*Farm: University of Illinois (UI), University of Massachusetts (UM), Private Owner (PO state), VA-MD Regional College of Veterinary Medicine (VM). BCS: (1-9) according to the Hennecke scale. BCS cat: 1-5.5 (Lean), 6-6.5 (Normal), 7+ (Obese). Feed: Pasture (P), Hay (H), Hay/Concentrate (HC). Age: 10 years or less (Y), and over 10 years (M)*.

### Blood sample collection

Whole blood was collected via venipuncture into untreated Vacutainer tubes (serum) and EDTA tubes (plasma) (BD, Franklin Lakes, NJ). Tubes were chilled for no more than 2 h before processing. Serum tubes were allowed to return to room temperature and clot before spinning. Plasma tubes were spun for 20 min at 850 g at 4°C. Serum tubes were spun for 20 min at 850 g at room temp. Plasma or serum layers were removed and stored at −80°C prior to analysis. All analysis was done at the Cornell Animal Health Diagnostic Center, Ithaca, NY. ACTH, cortisol, insulin, and leptin were measured from plasma samples, and glucose and triglycerides were measured from serum samples.

### DNA extraction and sequencing

Fresh fecal samples were collected midrectum from each horse, kept on ice for no more than 2 h prior to storage at −80°C. DNA was extracted utilizing either a modified CTAB-bead beating method (48–50), or Mobio Power Fecal DAN extraction kit (MoBio Laboratories, Carlsbad, CA), and stored at −80°C prior to sequencing.

Amplification of the V4-V5 region of the 16S rRNA gene and attachment of indexes for multiplexing samples were done using region specific primers (515F/926R) as described elsewhere (51). PCR products were pooled and sequenced using the MiSeq platform at either the University of Illinois Biotechnology Center, Urbana, IL, or RTL Genomics, Lubbock, TX. Paired ends were joined using FLASh (v. 1.2.11) (52). Quality and chimera filtering, taxonomic assignment, diversity analysis, and identification of shared and unique taxa were done using the QIIME (53) pipeline as applied previously (54).

### Statistical analysis

Relative abundance of bacterial groups and alpha diversity measures by body condition group were compared using pair-wise, two-tailed *t*-tests (assuming unequal variances), and Kruskal-Wallis rank sum test in R (55). Differential abundance between lean, normal, and obese horses at the taxa level was modeled using a negative binomial distribution in the DESeq package (56) in R (55). Spearman correlations of all pairs of taxa, blood analytes, metadata, and relative abundance of bacterial taxa were calculated in JMP (Pro 13.0.0).

### Network construction

Networks of significant Spearman correlations were visualized in Cytoscape (version 3.6.0). Taxa nodes were mapped to their phylogeny, colored by phyla, and assigned a two-letter code (**Table 3**). Border thickness of taxa nodes was proportional to Relative Abundance (RA). Significant positive and negative Spearman correlations were represented by red and blue edges, respectively. Edge thickness was proportional to correlation coefficient values ranging from +1 to +0.3 and from −1 to −0.3. Networks of nodes of differentially abundant taxa, were constructed by selecting first neighbors for all the specified nodes. In complex networks, edges representing pairwise correlations with values <0.5 were de-emphasized (faded).

## Results

### 16S rRNA sequencing

Summary statistics for 16S rRNA sequencing following filtering for low quality and length can be found in Table 2. The average read length was 412 bp, and the total number of reads was 3,148,220. Sequence data has been deposited in Genbank BioSample SAMN09917936.

**Table 2:** 16S rRNA sequence counts after removal of low quality and short reads.

**Counts/Sample summary**	
Number of samples	78
Minimum count	4165
Maximum count	102594
Median count	44498.5
Mean count	40361.795
Std. dev.	21151.638

### Bacterial abundance profiles

16S rRNA sequences were clustered at 97% similarity against the latest Greengenes database (13_5). The resulting operational taxonomic units (OTUs) were filtered for singletons and doubletons. Table 3 lists the 51 bacterial OTUs with abundance >0.10% with their corresponding 2 letter codes. All taxa included in the subsequent analysis are found in Table S2.

**Table 3:** Taxa identified and the total relative abundance in the obese, normal, and lean horse samples.

**Taxon lineage**	**Code**	**Relative abundance (%)**
Bacteria; Bacteroidetes; Bacteroidia; Bacteroidales;f__;g__	QE	16.244410
Bacteria; Firmicutes; Clostridia; Clostridiales;f__Ruminococcaceae;g__	LK	15.893878
Bacteria; Firmicutes; Clostridia; Clostridiales;f__Lachnospiraceae;g__	OJ	14.452446
Bacteria; Firmicutes; Clostridia; Clostridiales;f__;g__	QI	11.473584
Unassigned;Other;Other;Other;Other;Other	AA	3.780830
Bacteria; Firmicutes; Clostridia; Clostridiales;f__Ruminococcaceae;g__Ruminococcus	OK	3.094827
Bacteria; Firmicutes; Clostridia; Clostridiales;f__Mogibacteriaceae;g__	BL	2.678472
Bacteria; Spirochaetes; Spirochaetes; Spirochaetales;f__Spirochaetaceae;g__Treponema	FR	2.670410
Bacteria; Bacteroidetes; Bacteroidia; Bacteroidales;f__Prevotellaceae;g__Prevotella	DF	2.435187
Bacteria; Fibrobacteres; Fibrobacteria; Fibrobacterales;f__Fibrobacteraceae;g__Fibrobacter	EH	2.356017
Bacteria; Bacteroidetes; Bacteroidia; Bacteroidales;f__Paraprevotellaceae;g__CF231	KF	1.715601
Bacteria; Bacteroidetes; Bacteroidia; Bacteroidales;f__Paraprevotellaceae;g__YRC22	LF	1.563672
Bacteria; Firmicutes; Clostridia; Clostridiales;f__Lachnospiraceae;Other	NJ	1.352235
Bacteria; Firmicutes; Clostridia; Clostridiales;f__Veillonellaceae;g__Phascolarctobacterium	UK	1.251141
Bacteria; Bacteroidetes; Bacteroidia; Bacteroidales;f__Paraprevotellaceae;g__	JF	1.212778
Bacteria; Bacteroidetes; Bacteroidia; Bacteroidales;f__RF16;g__	EF	1.168953
Bacteria; Actinobacteria; Coriobacteriia; Coriobacteriales;f__Coriobacteriaceae;g__	YD	1.071085
Bacteria; Firmicutes; Clostridia; Clostridiales;f__Lachnospiraceae;g__Coprococcus	SJ	1.030847
Bacteria; Firmicutes; Clostridia; Clostridiales;f__Clostridiaceae;g__Clostridium	ZI	1.009161
Bacteria; Bacteroidetes; Bacteroidia; Bacteroidales;f__Bacteroidaceae;g__BF311	UE	0.882039
Bacteria; Firmicutes; Bacilli; Lactobacillales;f__Streptococcaceae;g__Streptococcus	MI	0.845326
Bacteria; Firmicutes; Clostridia; Clostridiales;f__Lachnospiraceae;g__Blautia	QJ	0.732944
Bacteria; Firmicutes; Clostridia; Clostridiales;f__Lachnospiraceae;g__Pseudobutyrivibrio	YJ	0.666919
Bacteria; Firmicutes; Clostridia; Clostridiales;f__Clostridiaceae;g__	VI	0.659299
Bacteria; Bacteroidetes; Bacteroidia; Bacteroidales;f__BS11;g__	RE	0.642893
Bacteria; Bacteroidetes; Bacteroidia; Bacteroidales;f__Paraprevotellaceae;g__Prevotella	MF	0.582616
Bacteria; Firmicutes; Clostridia; Clostridiales;f__Ruminococcaceae;g__Oscillospira	NK	0.550106
Bacteria; Actinobacteria; Coriobacteriia; Coriobacteriales;f__Coriobacteriaceae;g__Adlercreutzia	ZD	0.497567
Bacteria; Firmicutes; Clostridia; Clostridiales;f__Christensenellaceae;g__	TI	0.466590
Bacteria; Bacteroidetes; Bacteroidia; Bacteroidales;f__Porphyromonadaceae;g__Paludibacter	XE	0.443382
Bacteria; Proteobacteria; Alphaproteobacteria; f__;g__	MM	0.410118
Bacteria; Cyanobacteria; 4C0d-2; YS2;f__;g__	NG	0.404619
Bacteria; Tenericutes; Mollicutes; RF39;f__;g__	CS	0.361142
Bacteria; Firmicutes; Clostridia; Clostridiales;f__Mogibacteriaceae;g__Mogibacterium	DL	0.351654
Bacteria; Firmicutes; Erysipelotrichi; Erysipelotrichales;f__Erysipelotrichaceae;g__RFN20	WL	0.324342
Bacteria; Firmicutes; Clostridia;Clostridiales;f__Eubacteriaceae;g__Pseudoramibacter_Eubacterium	JJ	0.306375
Bacteria; Firmicutes; Clostridia; Clostridiales;Other;Other	PI	0.260408
Bacteria; Firmicutes; Erysipelotrichi; Erysipelotrichales;f__Erysipelotrichaceae;g__	PL	0.255276
Bacteria; Firmicutes; Clostridia; Clostridiales;f__Veillonellaceae;g__	RK	0.223319
Bacteria; Bacteroidetes; Bacteroidia; Bacteroidales;f__S24-7;g__	HF	0.217790
Bacteria; Firmicutes; Clostridia; Clostridiales;f__Lachnospiraceae;g__Dorea	TJ	0.214578
Bacteria; Firmicutes; Bacilli; Lactobacillales;f__Lactobacillaceae;g__Lactobacillus	KI	0.210029
Bacteria; Firmicutes; Clostridia; Clostridiales;f__Lachnospiraceae;g__Epulopiscium	UJ	0.202495
Bacteria; Firmicutes; Clostridia; Clostridiales;f__Clostridiaceae;Other	UI	0.190916
Bacteria; Firmicutes; Erysipelotrichi; Erysipelotrichales;f__Erysipelotrichaceae;g__p-75-a5	BM	0.185225
Bacteria; Spirochaetes; Spirochaetes; Sphaerochaetales;f__Sphaerochaetaceae;g__Sphaerochaeta	DR	0.170744
Bacteria; Firmicutes; Erysipelotrichi; Erysipelotrichales;f__Erysipelotrichaceae;g__Eubacterium	YL	0.160846
Bacteria; Firmicutes; Clostridia; Clostridiales;f__Lachnospiraceae;g__Ruminococcus	BK	0.157950
Bacteria; Bacteroidetes; Bacteroidia; Bacteroidales;f__Bacteroidaceae;g__Bacteroides	VE	0.122171
Bacteria; Firmicutes; Clostridia; Clostridiales;f__Lachnospiraceae;g__Roseburia	ZJ	0.119148
Bacteria; Tenericutes; Mollicutes; Anaeroplasmatales;f__Anaeroplasmataceae;g__Anaeroplasma	AS	0.106584

*Two-letter codes designations used in the network analysis and total relative abundance over 0.10% are shown. For all taxa, see Table S2*.

**Figure 1:**
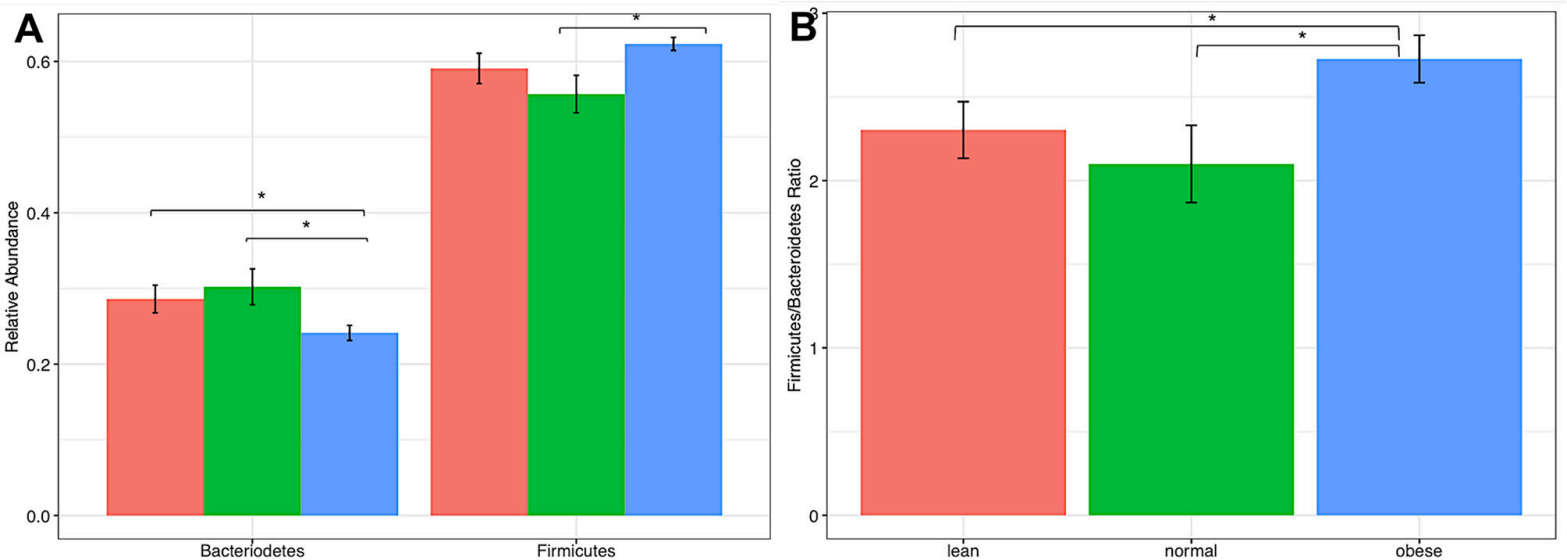
Comparison of firmicutes and bacteriodetes from lean, normal, and obese horses. **(A)** Relative abundance of each Phla. **(B)** Firmicutes /Bacteriodetes ratio. Error bars represent standard error. Significantly different groups (two tailed *t*-test assuming unequal variances) art indicated*.

At the phyla level, comparison of communities of lean, obese, and normal horses showed no significant differences in variance (Kruskal-Wallis test, *p*-value > 0.05) (Figure S1), however pairwise differences were detected between obese and lean and obese and normal horses in relative abundance of Bacteroidetes and Firmicutes (two-tailed *t*-test assuming unequal variances, *p*-value < 0.05) (Figure 1). Specifically, the relative abundance of Bacteroidetes was less in obese horses, while the relative abundance of Firmicutes was higher. Consequently, the Firmicutes/Bacteroidetes ratio was higher for obese horses. Comparison of Bacteroidetes families in the gut microbiome of obese, lean, and normal horses show differences in unspecified Bacteroidales family and Porphyromonadaceae, while difference were seen in six Firmicutes families: Christensenellaceae, Erysipelotrichaceae, Lachnospiraceae, Lactobacillaceae, Mogibacteriaceae, and Ruminococcaceae (Figure S2).

**Figure 2:**
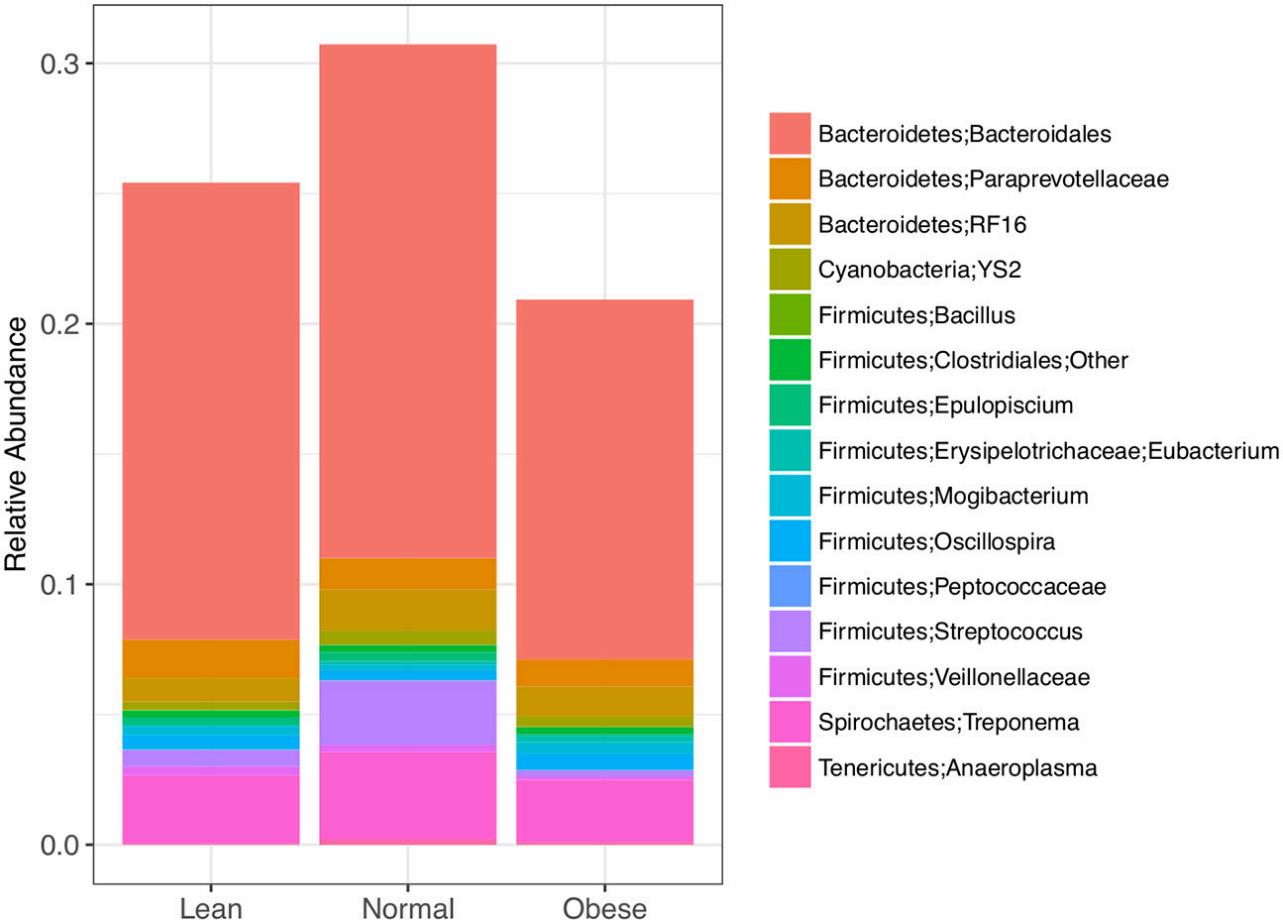
Differently abundant bacterial taxa between lean, normal, and obese horses as determined using a negative binomial distribution, *p-*value <0.05. Taxa with relative abundance ≥ 0.01 % are shown.

Differentially abundant taxa were identified (padj < 0.05) using a negative binomial distribution in DESeq for pairwise BCS groups and All BCS groups together (Table 4). All but four differentially abundant taxa were members of Actinobacteria, Firmicutes, or Bacteroidetes. There were 5, 6, and 24 differentially abundant taxa between Obese/Lean, Normal/Lean, and Obese/Normal groups respectively. Nine taxa were found to be differentially abundant in two or more pair-wise comparisons, and three taxa were identified as differentially abundant in comparisons of all BCS categories. Differentially abundant taxa with relative abundance >0.01% were compared by BCS group (Figure 2), and found to collectively constitute between 20 and 30% of total bacterial abundance.

**Table 4:** Differentially abundant taxa.

**Taxa lineage**	**Code**	**Number of connections**	**Relabund%**	**Obese/Lean**	**Obese/Normal**	**Lean/Normal**	**All BSC**
Firmicutes;Clostridiaceae;SMB53	AJ	29	0.01		X		
Anaeroplasmataceae;Anaeroplasma	AS	10	0.11		X	X	
Actinobacteria;Coriobacteriaceae;Collinsella	BE	74	0.01		X		
Actinobacteria; Microbacteriaceae;Microbacterium	CC	109	0.01		X	X	
Firmicutes;Peptococcaceae;g__	CK	56	0.03		X		
Firmicutes; Mogibacteriaceae;Mogibacterium	DL	76	0.35		X		
Bacteroidetes;Bacteroidales;RF16	EF	23	1.17		X		
Fibrobacteraceae;Fibrobacter	EH	9	2.36		X		
Firmicutes;Lactobacillales;Other	EI	19	0.00		X		
Firmicutes;Clostridiales;f__EtOH8	EJ	5	0.01		X		
Spirochaetaceae;Treponema	FR	15	2.67		X		
Firmicutes;Peptostreptococcaceae;g__	HK	38	0.01		X		
Verrucomicrobia;RFP12;g__	HS	23	0.03	X	X		X
Actinobacteria;Micrococcaceae;g__	IC	102	0.03			X	
Bacteroidetes;Paraprevotellaceae;Other	IF	17	0.01		X		
Bacteroidetes;Paraprevotellaceae;g__	JF	19	1.21	X	X		X
Firmicutes;Bacillaceae;Bacillus	JH	88	0.02		X	X	
Firmicutes;Streptococcaceae;Streptococcus	MI	27	0.85		X		
Cyanobacteria;YS2;f__;g__	NG	25	0.40		X		
Firmicutes;Ruminococcaceae;Oscillospira	NK	65	0.55		X	X	
Firmicutes;Clostridiales;Other;Other	PI	3	0.26		X		
Bacteroidetes;Bacteroidales;f__;g__	QE	69	16.24	X	X		
Firmicutes;Veillonellaceae;g__	RK	74	0.22	X	X		X
Actinobacteria;Nocardiaceae;Rhodococcus	TC	119	0.01		X	X	
Firmicutes;Lachnospiraceae;Epulopiscium	UJ	28	0.20		X		
Firmicutes;Erysipelotrichaceae;Eubacterium	YL	48	0.16	X			X

*Taxa identified in whole group and pairwise comparisons (padj < 0.05) using linear binomial distribution in DESeq. Network connectivity is estimated by numbers of connections in correlation network*.

### Bacterial diversity

**Figure 3:**
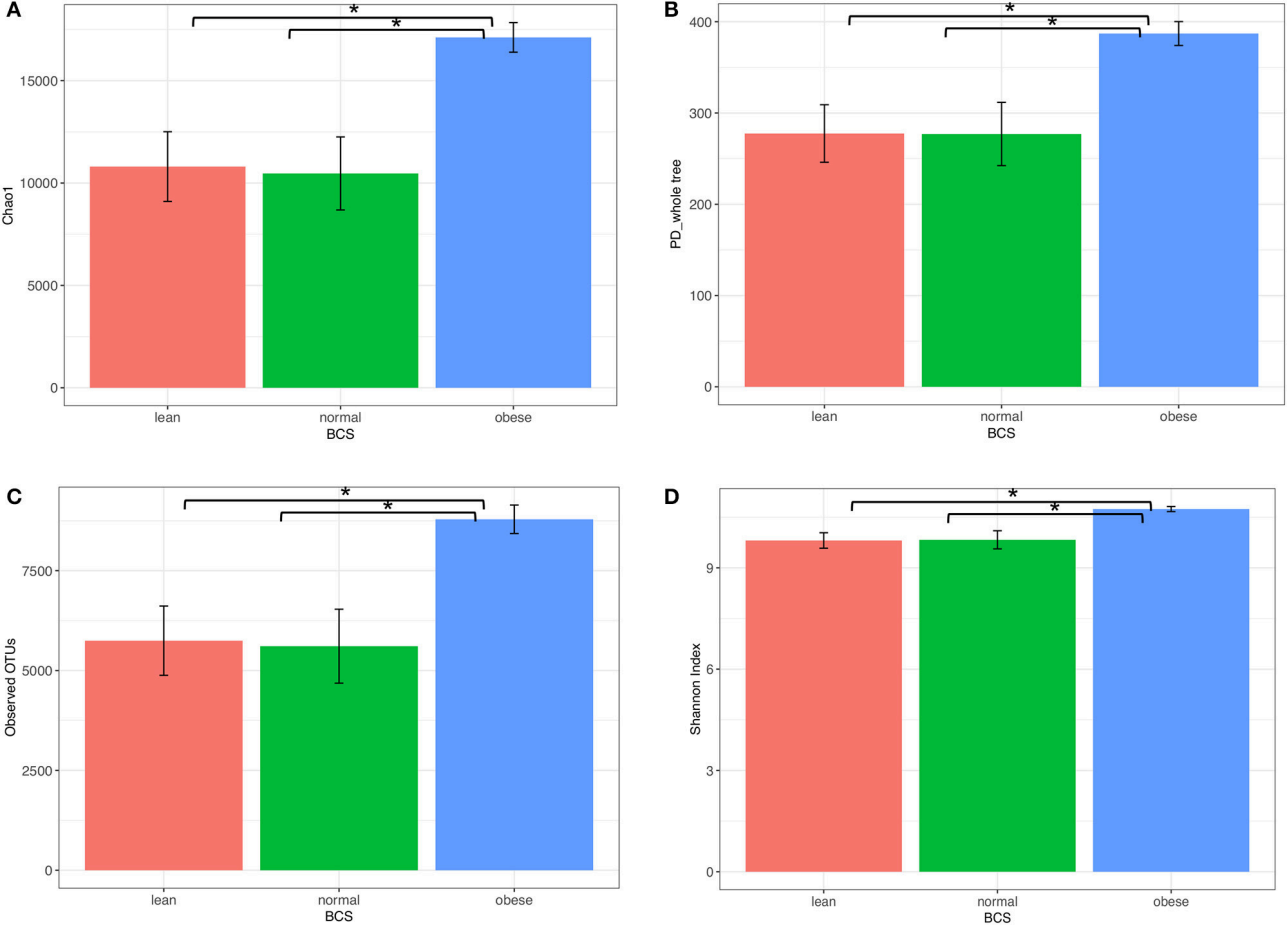
Alpha diversity of bacterial communities from obese, normal, and lean horses. **(A)** chaol, **(B)** PD_whole _tree, **(C)** observed OTUs, **(D)** Shannon Index. Error bars represent standard error. Significantly different groups (two tailed *t*-test assuming unequal variances) art indicated*.

Obese horse samples were higher than both normal and lean for all measures of alpha diversity, including richness (Chao1 and Observed OTUs), richness and evenness (Shannon Index), and phylogenetic diversity (PD-whole-Tree) (Figure 3).

### Blood analytes

**Figure 4:**
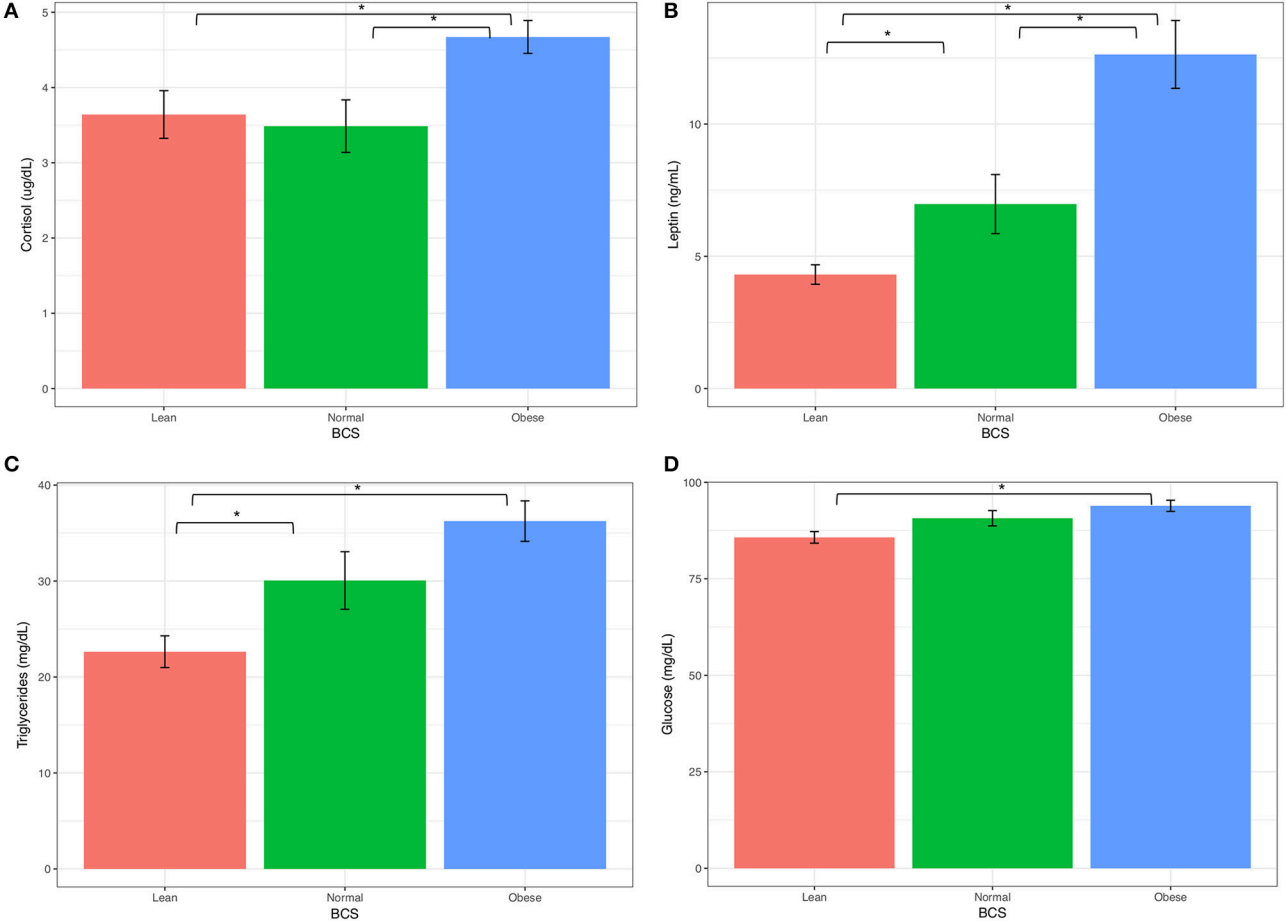
Keyboard analytes from obese, normal, and lean horses **(A)** Corsitol, **(B)** Leptin, **(C)** Triglycerides, **(D)** Glucose. Error bars represent standard error. Significantly different groups (two tailed *t*-test assuming unequal variances) art indicated*.

Measurements of insulin, glucose, ACTH, cortisol, leptin, and triglycerides were measured from either serum or plasma, and summarized by BCS group in Table 5. All blood analytes by horse are reported on Table S3. Blood levels of cortisol were higher for obese horses than normal or lean horses. Levels of leptin increased with increasing BCS. Triglyceride and glucose levels were similar between normal and obese horses, and lower for lean horses (Figure 4). Statistical difference was not seen between horse groups for resting insulin or ACTH (not shown).

**Table 5:** Summary of blood analyte measurements based on BCS category.

	**Lean** ***n** = **24***		**Normal** ***n** = **17***		**Obese** ***n** = **37***	
**Measurement**	**Mean**	**SD**	**Mean**	**SD**	**Mean**	**SD**
BCS	4.56	0.60	6.16	0.24	7.46	0.48
Insulin (uIU/ml)	10.78	8.64	15.74	9.76	15.49	16.33
ACTH (pg/ml)	21.53	8.84	20.21	7.12	24.21	10.63
Cortisol (ug/dL)	3.73	1.55	3.49	1.44	4.67	1.33
Leptin (ng/ml)	4.32	1.94	6.56	4.64	12.63	7.59
Glucose (mg/dL)	86.00	7.39	90.71	8.14	93.92	8.80
Triglycerides (mg/dL)	23.26	7.93	30.06	12.36	36.24	12.84

**Figure 5:**
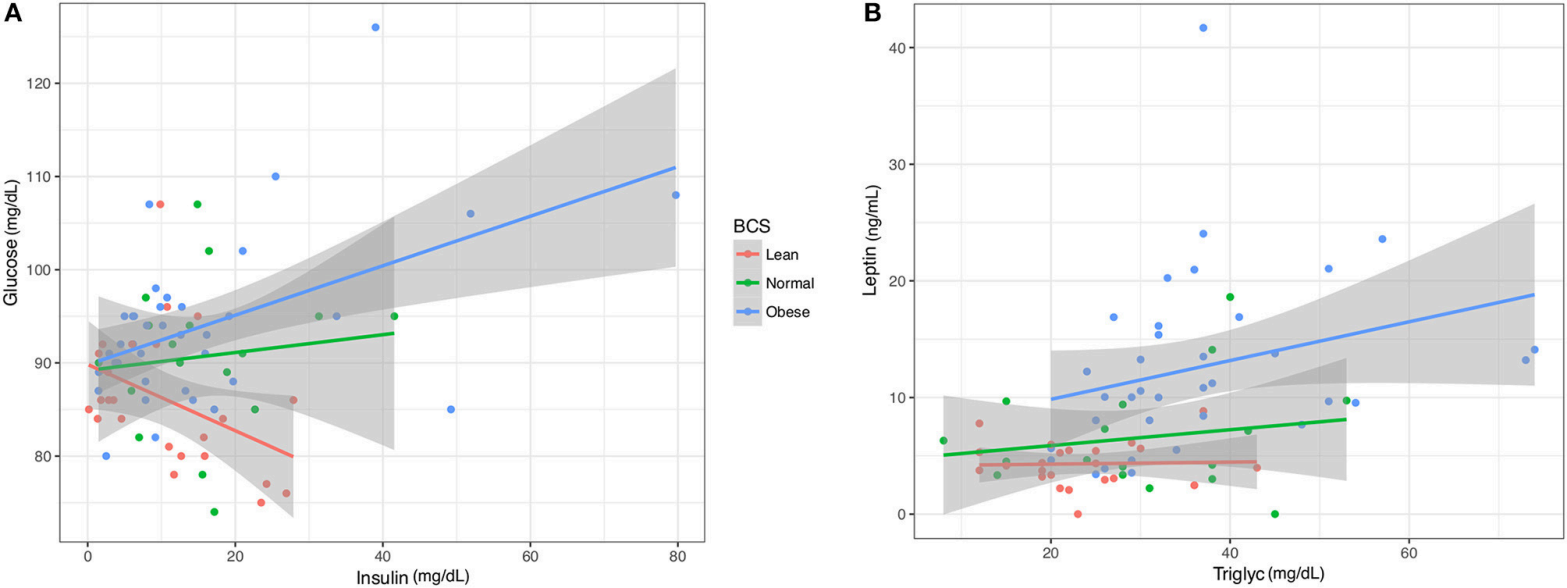
Linear models of key blood analytes from obese, normal, and lean horses. **(A)** Insulin vs. Glucose. **(B)**. Triglycerides vs. Leptin. Gray region represents 95% confidence intervals.

The relationships between pairs of blood factors was plotted with 95% confidence intervals to identify patterns based on BCS (Figure 5). At the ranges measured, clear differences were seen in the trend for insulin and glucose in obese, normal, and lean horses. A positive slope for obese and normal samples showed that glucose and insulin levels increased proportionally. An opposite trend was shown for lean horses, as glucose dropped with increasing insulin levels. There was no overlap between confidence intervals for lean and either normal or obese horses. Between normal and obese horses, overlap occurred for only the upper confidence interval. Linear modeling of triglycerides and leptin showed a more positive relationship and leptin response in the obese horses, and nearly constant leptin levels in normal and lean horses. Confidence intervals did not overlap between the obese group and either the lean or normal horses, which were more consistent with each other.

### Correlation analysis

Spearman rank correlation coefficients analysis performed in JMP (Pro 13.0.0) or R (55) included all 446 taxa, six blood analytes and four metadata variables: Feed, Age, BCS, and Owner. The default alpha value for the initial pairwise analysis was 0.05. 105,570 correlations were found. Correlations with *p*-values ≤ 0.01 and coefficient values in the range of −0.3 to −1.0 and +0.3 to +1.0 resulted in 9,353 significant pairwise interactions for network analysis.

### Network analysis

**Figure 6:**
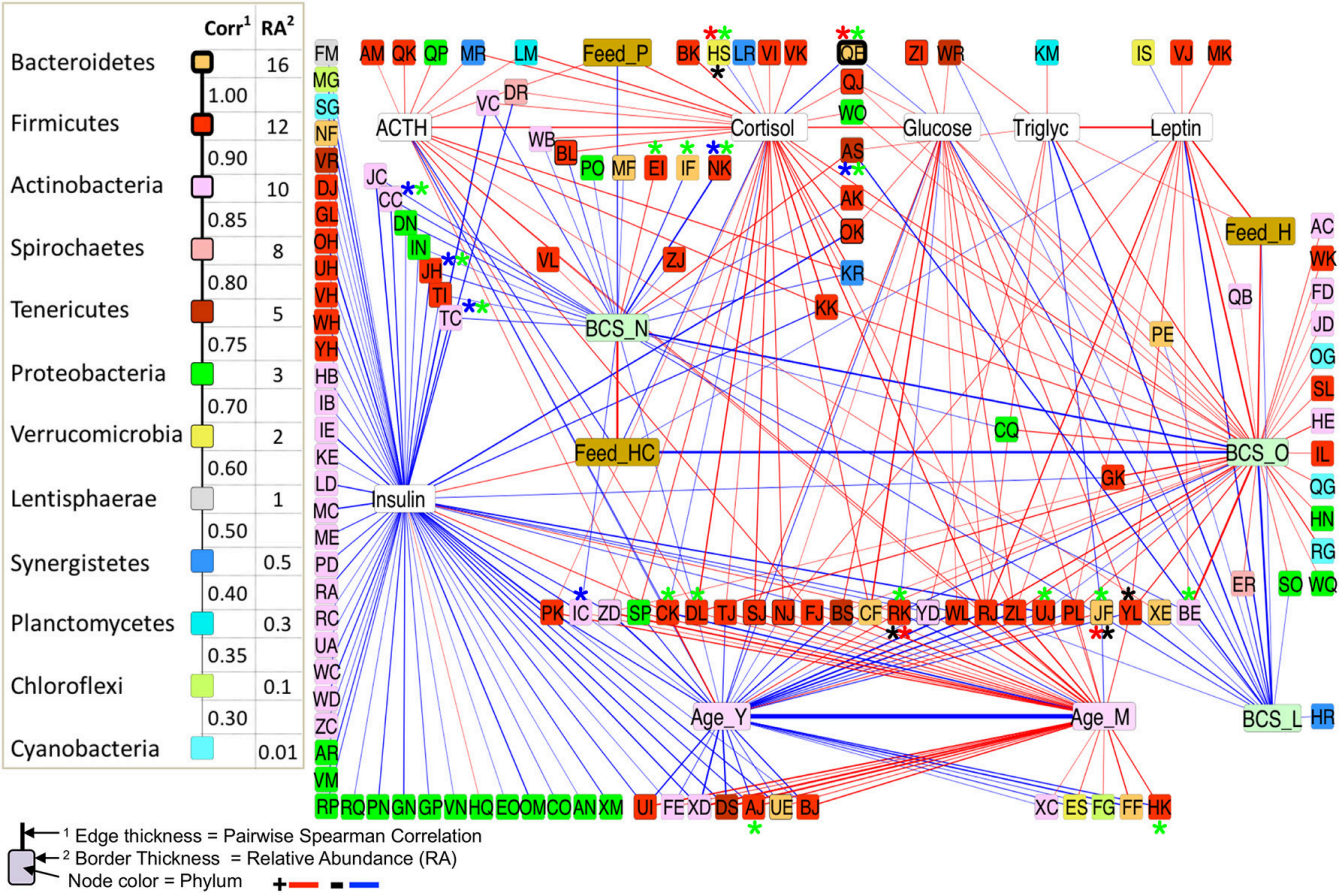
First-neighbors networks of significant pairwise correlations for body condition score, blood analytes and age with differentially abundant (DA) taxa highlighted. Among All BCS*, between L/O horses*, L/N horses*, and O/N horses*.

Networks of significant Spearman correlations were visualized in Cytoscape (version 3.6.0). This step resulted in a network composed of 458 nodes and 9,353 edges. The first neighbor network, showing significant correlations between all blood analytes, metadata, and taxa (Figure 6), showed positive correlations between BCS_O (obese) and blood analytes leptin, cortisol, triglycerides, and glucose, but no correlation with ACTH or insulin. BCS_O was positively correlated with Feed_H (hay), negatively correlated with Feed_HC (hay-concentrate), and not connected with Feed_P (pasture). Focusing on the differentially abundant taxa, the microbial network positively associated with BCS_O included 32 taxa, primarily from Actinobacteria, Firmicutes, and Bacteroidetes. BCS_O had only a few negatively associated bacteria, including highly connected members of the Veillonellaceae (RK), and Lachnospiraceae (UJ).

BCS_L (lean) showed negative correlations with leptin, glucose, and triglycerides, and no correlation with any feed group. The microbial network negatively associated with BCS_L included taxa positively associated with BCS_O or BCS_N, specifically Anaeroplasma (AS), Eubacterium (JF), and Paraprevotellaceae (YL).

BCS_N (normal) was not connected to any blood analyte, but showed positive correlation to Feed_HC and negative correlation to Feed_H. Negative correlations were shown for twenty taxa, including differentially abundant Oscillospira (NK), Microbacterium (CC), Bacillus (JH), and Rhodococcus (TC).

Each of the blood analytes had a small sub-network of associations, except insulin, which showed negative correlations with over 50 bacterial taxa, and no connection to BCS. Insulin did show a positive correlation to Feed_HC, and a negative correlation with Age_Y (young).

**Figure 7:**
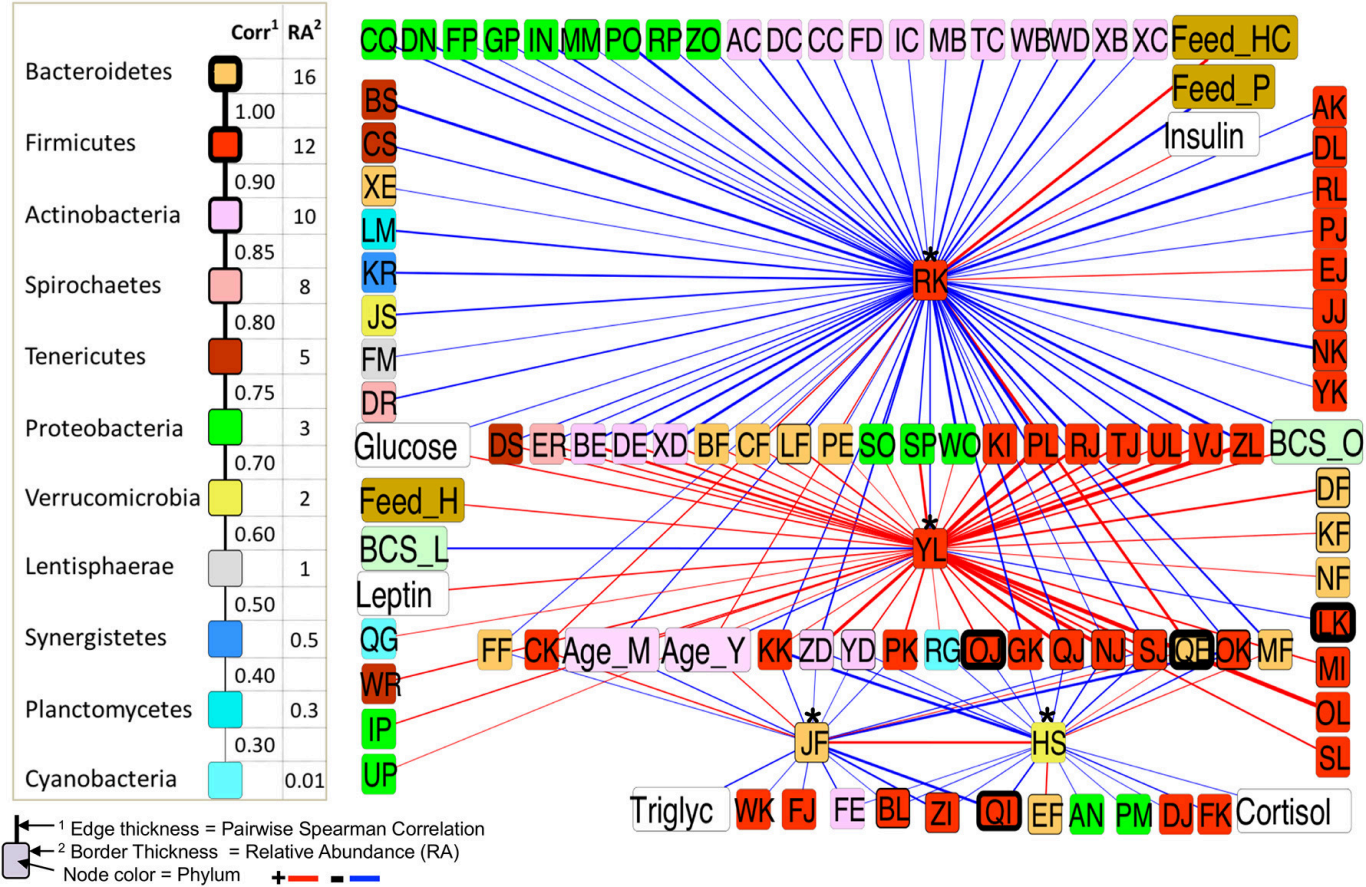
First-neighbor network for Spearman significant pairwise correlations of all differentially abundant (DA) taxa among all BCS categories.

The first neighbor network of the differentially abundant taxa for all BCS groups (Figure 7) showed the connectivity of these four taxa. Veillonellaceae (RK) was positively correlated with a Bacteroidetes (QE), a highly abundant (16.24%) taxa in the dataset, but negatively associated with 24 taxa that were all positively associated with an Erysipelotrichaceae (YL), suggesting a strong relationship between these two taxa. RK was also positively associated with insulin and Feed_HC, and negatively correlated with glucose and Feed_P, while YL was positively correlated with glucose, leptin, Feed-H, BCS_O, and Age_M (middle aged), and negatively associated with Age_Y and BCS_L.

**Figure 8:**
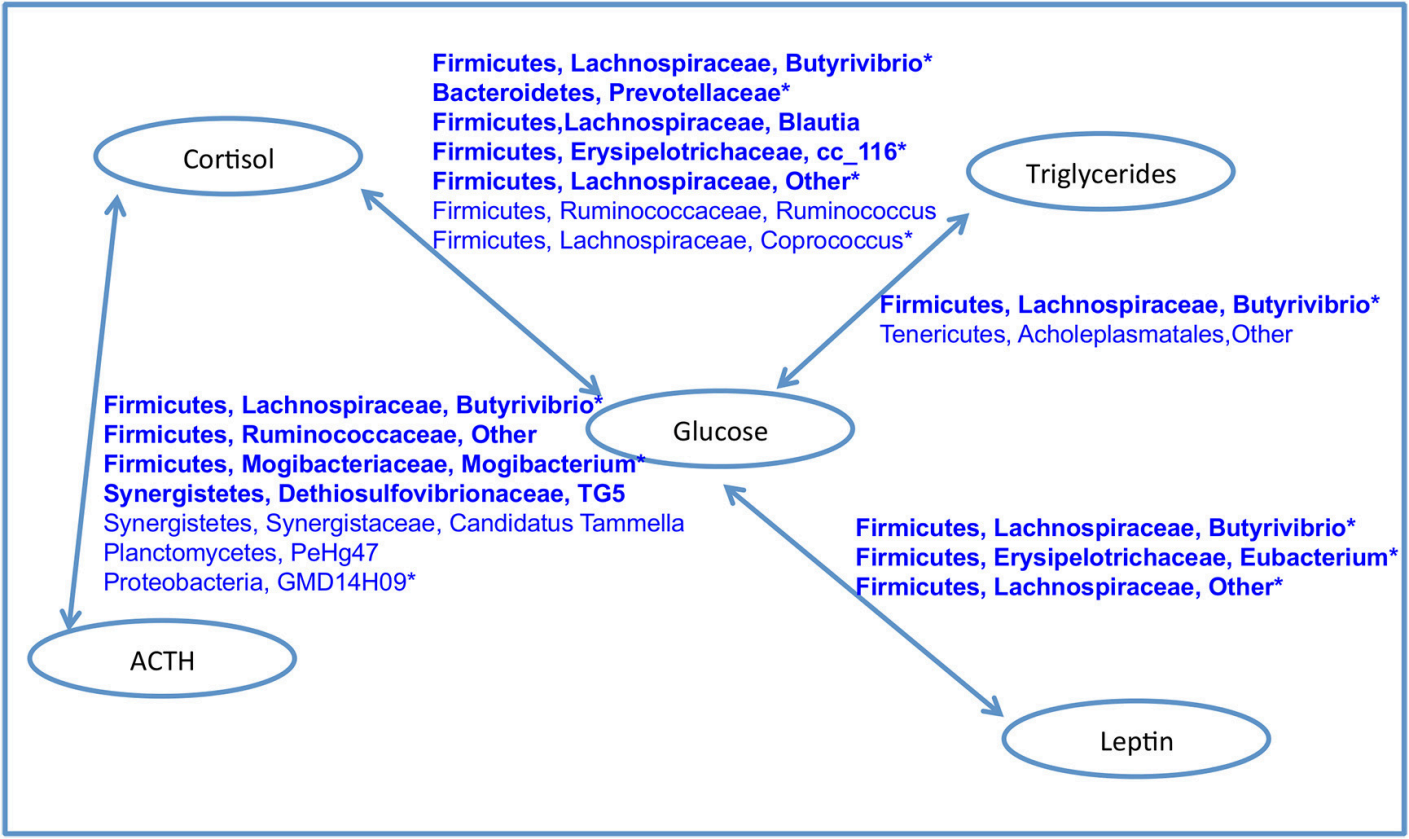
Network of bacteria with positive associations with both blood analytes in each connected pair. Taxa in bold were positively correlated with BCS_O. Starred taxa were positively associated with the older age group. No bacterial taxa was positively associated with BCS_O, BCS_N and any pair of analytes. No bacterial taxa was positively with insulin and any other analyte.

A network of bacteria containing only positive correlations with two or more blood analytes points to key taxa which are also associated with BCS_O and the older age group (Figure 8). This group contained nine Firmicutes, two Synergistetes, and one each of Bacteroidetes, Planctomycetes, and Proteobacteria. Of special interest were two taxa: *Firmicutes, Lachnospiraceae, Butyrivibrio*, and *Firmicutes, Lachnospiraceae, Other* which were positively correlated to two and four pairs of associations respectively.

### Analysis of additional metadata factors

Significant correlations were found between owner and feed type, but not owner and BCS or any other blood analyte presumably due to consistence in management methods between farms. Three taxa were found to be uniquely correlated with owner: two Bacteroidetes (Rikenellaceae and Paraprevotellaceae, *YRC22*), and a Firmicutes (*Streptococcus* spp.) (Table 6). These were found in the dataset at 1.56, 0.012, and 0.845% respectively.

**Table 6:** Spearman correlations between owner, metadata factors and bacteria.

**Factor**	**Correlation**
Age	0.164
Feed	0.508^*^
BCS	0.294
Insulin	0.113
ACTH	−0.135
Cortisol	−0.110
Leptin	−0.223
Glucose	0.176
Triglycerides	−0.007
Bacteroidetes,Paraprevotellaceae,YRC22 (LF)	0.332^*^
Bacteroidetes,Rikenellaceae (FF)	0.332^*^
Firmicutes,Lactobacillales,Streptococcus (MI)	−0.367^*^

Only bacteria uniquely correlated with owner are shown. Significant correlations (coefficients >0.3 or <-0.3) are indicated ^*^.

## Discussion

This research compares the diversity and structure of gut microbiome communities of obese, lean, and normal horses, and correlates bacterial community assembly with blood analytes associated with obesity and metabolic issues in horses. The blood marker results (higher leptin, triglycerides, glucose, and cortisol levels, and trends toward higher insulin in obese horses) mirror what has been shown in other studies (45, 47, 57), but this is the first report correlating BCS, blood analytes, and microbial community composition in horses.

Similar to surveys of obese individuals in other systems, we report higher phylogenetic diversity and greater richness of bacteria in the gut microbiomes of the BCS_O horses (40, 41, 58). Specific Firmicutes groups (members of the Ruminococcaceae and Lachnospiraceae families) were positively correlated with two or more key blood analytes, increasing age, and obesity (Figure 8). While collectively this highly connected network of bacteria comprises <5% of the relative abundance of sequences in the data set, they could be providing beneficial metabolic products and ecosystem services.

We report obese BCS in horses to be positively correlated to four blood analytes: glucose, cortisol, triglycerides, and leptin, and lean BCS to be negatively correlated to glucose, tryglycerides, and leptin. These values were similar to prior studies in horses (27, 59, 60), and have been used in diagnostic panels for EMS. In humans, it has been estimated that the gut microbiome could explain 4.5–6% of the variation in BMI and triglyceride levels (61), specifically 114 taxa, 95 of which were members of Firmicutes (Lachnospiraceae, Ruminococcaceae, Christensenellaceae, and others). While horses typically consume a relatively low fat diet, obese BCS gut microbiomes were found to be enriched in six triglyceride associated bacterial taxa, while the lean BCS group was not positively correlated with any of these taxa. Specific obesity related taxa from human studies were positively associated with obese BCS in this study, in particular *Campylobacter* spp., *Collinsella* spp., Prevotellaceae, *Selenomonas* spp., *Blautia* spp., and *Mogibacterium* spp. (62, 63), three taxa of Cyanobacteria, and *Adlercreutzi* spp. (64), four Erysipelotrichaceae taxa associated with obesity (65) and aromatic amino acid metabolism in high fat diet (66), and Dethiosulfovibrionaceae, a family of sulfate reducing bacteria (64, 66–68). That the normal and lean BCS groups were either negatively or not correlated with all of these taxa suggests distinguishing community differences in horses based on BCS, and points to similarities in host-microbial dynamics underlying metabolic disease between horses and humans.

At the same time, four taxa associated with healthy gut status were significantly correlated with obese BCS, specifically Propionibacteriaceae (propionate producer), *Butyrivibrio* spp. (butyrate producer), Ruminococcaceae (fiber degrader), and *Sutterella* spp. (function unclear) (62). *Butyrivibrio* spp. was of special interest because it was significantly correlated with all four pairs of blood analytes (Figure 8). While its abundance is <1% in the dataset, the high connectivity of this bacteria suggests that it could play an important role in host interactions, regulation, or immune status related to obesity.

The lack of correlation between resting insulin and bacterial taxa abundance found in this study reflected the difficulty in estimating blood insulin values using a resting measurement (27, 46), or suggested a more complex picture. Horses with high blood insulin and glucose levels are often, but not always obese (27, 39, 47). A more complete model of the gut microbiome and insulin dynamics would be possible by comparing the microbiomes of both lean and obese horses with a wider range of insulin levels.

While gut microbiome differences were seen in horses based on diet, it was not possible to associate feed with BCS as it is a driver for management decisions, especially given the relatively small numbers of owners and the consistency of their feeding patterns. The obese BCS horses were largely being fed hay or pasture only, and the lean and normal BCS horses were consuming hay/concentrate, resulting in a significant association between owner and feed (Table 6). Significant correlations were also noted based on age, but were inconclusive since the categories were broadly divided and included no horse above 20 years. Managing older horses will continue to be a challenge in the future as the numbers of aged horses increases, therefore future work to identify bacteria correlated with obesity and blood markers associated with age-related metabolic issues is warranted.

This research points to differences in the gut microbiomes of lean, normal, and obese horses that are significantly correlated to key blood analytes associated with BCS. Network analysis points to signature species for each body condition category, laying the foundation for experiments leading to a mechanistic understanding, and more targeted microbial solutions to the issue of obesity and metabolic syndrome in horses.

## Ethics statement

This study was carried out in accordance with the recommendations of the USDA Animal Welfare Act and the NIH Public Health Service Policy on the Humane Care and Use of Animals, University of Illinois Institutional Animal Care and Use Committee (IACUC). The protocol was approved by the University of Illinois Institutional Animal Care and Use Committee (IACUC).

## Author contributions

AB obtained and prepared blood and fecal samples, gained funding, analyzed 16S rRNA sequence data, and wrote the manuscript. J-FT and ZF performed the pairwise correlation analysis in JMP and prepared the Cytoscape networks.

### Conflict of interest statement

The authors declare that the research was conducted in the absence of any commercial or financial relationships that could be construed as a potential conflict of interest.

## References

[1] Argenzio RA Southworth M Stevens CE. Sites of organic acid production and absorption in the equine gastrointestinal tract. Am J Physiol. (1974) 226:1043–50. 10.1152/ajplegacy.1974.226.5.1043 4824856

[2] Glinsky MJ Smith RM Spires HR Davis CL. Measurement of volatile fatty acid production rates in the cecum of the pony. J Anim Sci. (1976) 42:1465–70. 10.2527/jas1976.4261465x 931822

[3] Costa MC Arroyo LG Allen-Vercoe E Stämpfli HR Kim PT Sturgeon A. Comparison of the fecal microbiota of healthy horses and horses with colitis by high throughput sequencing of the V3-V5 region of the 16S rRNA gene. PLoS ONE (2012) 7:e41484. 10.1371/journal.pone.0041484 22859989 PMC3409227

[4] Costa MC Silva G Ramos RV Staempfli HR Arroyo LG Kim P. Characterization and comparison of the bacterial microbiota in different gastrointestinal tract compartments in horses. Vet J. (2015) 205:74–80. 10.1016/j.tvjl.2015.03.018 25975855

[5] Dougal K Harris PA Edwards A Pachebat JA Blackmore TM Worgan HJ. A comparison of the microbiome and the metabolome of different regions of the equine hindgut. FEMS Microbiol Ecol. (2012) 82:642–52. 10.1111/j.1574-6941.2012.01441.x 22757649

[6] Dougal K de la Fuente G Harris PA Girdwood SE Pinloche E Newbold CJ. Identification of a core bacterial community within the large intestine of the horse. PLoS ONE (2013) 8:e77660. 10.1371/journal.pone.0077660 24204908 PMC3812009

[7] Shepherd ML Swecker WS Jensen RV Ponder MA. Characterization of the fecal bacteria communities of forage-fed horses by pyrosequencing of 16S rRNA V4 gene amplicons. FEMS Microbiol Lett. (2012) 326:62–8. 10.1111/j.1574-6968.2011.02434.x 22092776

[8] Daly K Proudman CJ Duncan SH Flint HJ Dyer J Shirazi-Beechey SP. Alterations in microbiota and fermentation products in equine large intestine in response to dietary variation and intestinal disease. Br J Nutr. (2011) 107:989–95. 10.1017/S0007114511003825 21816118

[9] Dougal K de la Fuente G Harris PA Girdwood SE Pinloche E Geor J. (2014). Characterisation of the faecal bacterial community in adult and elderly horses fed a high fibre, high oil or high starch diet using 454 pyrosequencing. PLoS ONE 9:e87424. 10.1371/journal.pone.0087424 24504261 PMC3913607

[10] de Fombelle A Julliand V Drogoul C Jacotot E Characterization of the microbial and biochemical profile of the different segments of the digestive tract in horses given two distinct diets. Acta Agric Scand Secion Anim Sci. (2003) 77:293–304. 10.1017/S1357729800059038

[11] Shirazi-Beechey SP. Molecular insights into dietary induced colic in the horse. Equine Vet J. (2008) 40:414–21. 10.2746/042516408X314075 18487108

[12] Elzinga SE Weese JS Adams AA Comparison of the fecal microbiota in horses with equine metabolic syndrome and metabolically normal controls fed a similar all-forage diet. J Equine Vet Sci. (2016) 44:9–16. 10.1016/j.jevs.2016.05.010

[13] de Fombelle A Julliand V Drogoul C Jacotot E Feeding and microbial disprders in horses: 1-Effects of an abrupt incorporation of two levels of barley in a hay diet on microbial profile and activities. J Equine Vet Sci. (2001) 21:439–45. 10.1016/S0737-0806(01)70018-4

[14] Muhonen S Julliand V Lindberg JE Bertilsson J Jansson A. Effects on the equine colon ecosystem of grass silage and haylage diets after an abrupt change from hay. J Anim Sci. (2009) 87:2291–8. 10.2527/jas.2008-1461 19329474

[15] Louis P Scott KP Duncan SH Flint HJ. Understanding the effects of diet on bacterial metabolism in the large intestine. J Appl Microbiol. (2007) 102:1197–208. 10.1111/j.1365-2672.2007.03322.x 17448155

[16] Respondek F Goachet AG Julliand V. Effects of dietary short-chain fructooligosaccharides on the intestinal microflora of horses subjected to a sudden change in diet. J Anim Sci. (2007) 86:316–23. 10.2527/jas.2006-782 17940163

[17] Elzinga S Nielsen BD Schott HC Rapson J Robison CI McCutcheon J Comparison of nutrient digestibility between adult and aged horses. J Equine Vet Sci. (2014) 34:1164–9. 10.1016/j.jevs.2014.06.021

[18] Park K-D Park J Ko J Kim BC Kim H-S Ahn K. Whole transcriptome analyses of six thoroughbred horses before and after exercise using RNA-Seq. BMC Genomics (2012) 13:473. 10.1186/1471-2164-13-473 22971240 PMC3472166

[19] Willing B VöRöS A Roos S Jones C Jansson A Lindberg JE. Changes in faecal bacteria associated with concentrate and forage-only diets fed to horses in training. Equine Vet J. (2009) 41:908–14. 10.2746/042516409X447806 20383990

[20] Robin CA Ireland JL Wylie CE Collins SN Verheyen KLP Newton JR. Prevalence of and risk factors for equine obesity in Great Britain based on owner-reported body condition scores: prevalence of and risk factors for equine obesity in Great Britain. Equine Vet J. (2015) 47:196–201. 10.1111/evj.12275 24735219

[21] Thatcher CD Pleasant RS Geor RJ Elvinger F Negrin KA Franklin J Prevalence of obesity in mature horses: an equine body condition study. J Anim Physiol Anim Nutr. (2008) 92:222 10.1111/j.1439-0396.2007.00789_8.x

[22] Thatcher CD Pleasant RS Geor RJ Elvinger F. Prevalence of overconditioning in mature horses in southwest virginia during the summer. J Vet Intern Med. (2012) 26:1413–8. 10.1111/j.1939-1676.2012.00995.x 22946995

[23] Wyse A McNie KA Tannahil VJ Love S Murray JK. Prevalence of obesity in riding horses in Scotland. Vet Rec. (2008) 162:590–1. 10.1136/vr.162.18.590 18453379

[24] Henneke DR Potter GD Kreider JL Yeates BF. Relationship between condition score, physical measurements and body fat percentage in mares. Equine Vet J. (1983) 15:371–2. 10.1111/j.2042-3306.1983.tb01826.x 6641685

[25] Slater J National Equine Health Survey (NEHS) 2017. London: Royal Veterinary College (2017).

[26] Frank N Insulin resistance in horses. In: American Association of Equine Practitioners Proceedings. Seattle, WA (2006). 51–4.

[27] Frank N Elliott SB Brandt LE Keisler DH. Physical characteristics, blood hormone concentrations, and plasma lipid concentrations in obese horses with insulin resistance. J Am Vet Med Assoc. (2006) 228:1383–90. 10.2460/javma.228.9.1383 16649943

[28] Kronfeld DS Treiber KH Hess TM Boston RC Insulin resistance in the horse: definition, detection, and dietetics. J Anim Sci. (2005) 83:E22–31. 10.2527/2005.8313_supplE22x 16160047

[29] Vick MM Adams AA Murphy BA Sessions DR Horohov DW Cook RF. Relationships among inflammatory cytokines, obesity, and insulin sensitivity in the horse. J Anim Sci. (2007) 85:1144–55. 10.2527/jas.2006-673 17264235

[30] Walsh DM McGowan CM McGowan T Lamb SV Schanbacher BJ Place NJ Correlation of plasma insulin concentration with Laminitis Score in a field study of Equine Cushing's Disease and Equine Metabolic Syndrome. J Equine Vet Sci. (2009) 29:87–94. 10.1016/j.jevs.2008.12.006

[31] Agne B Laminitis: recognition of at-risk individuals, and methods of prevention. J Equine Vet Sci. (2010) 30:471–4. 10.1016/j.jevs.2010.07.012

[32] Carter RA Treiber KH Geor RJ Douglass L Harris PA. Prediction of incipient pasture-associated laminitis from hyperinsulinaemia, hyperleptinaemia and generalised and localised obesity in a cohort of ponies. Equine Vet J. (2009) 41:171–8. 10.2746/042516408X342975 19418747

[33] Cymbaluk NF Christison GI. Environmental effects on thermoregulation and nutrition of horses. Vet Clin North Am Equine Pract. (1990) 6:355–72. 10.1016/S0749-0739(17)30546-1 2202497

[34] Johnson PJ Wiedmeyer CE Ganjam VK. medical implications of obesity in horses—lessons for human obesity. J Diabetes Sci Technol. (2009) 3:163–74. 10.1177/193229680900300119 20046661 PMC2769846

[35] Kearns CF Mckeever KH Kumagai K Abe T. Fat-free mass is related to one-mile race performance in Elite standardbred horses. Vet J. (2002) 163:260–6. 10.1053/tvjl.2001.0656 12090768

[36] Kuyinu EL Narayanan G Nair LS Laurencin CT. Animal models of osteoarthritis: classification, update, and measurement of outcomes. J Orthop Surg. (2016) 11:19. 10.1186/s13018-016-0346-5 26837951 PMC4738796

[37] Norrdin RW Kawcak CE Capwell BA McIlwraith CW. Subchondral bone failure in an equine model of overload arthrosis. Bone (1998) 22:133–9. 10.1016/S8756-3282(97)00253-6 9477236

[38] Bamford NJ Potter SJ Baskerville CL Harris PA Bailey SR Effect of increased adiposity on insulin sensitivity and adipokine concentrations in different equine breeds adapted to cereal-rich or fat-rich meals. Vet J. (2016) 214:14–20. 10.1016/j.tvjl.2016.02.002 27387720

[39] Pleasant RS Suagee JK Thatcher CD Elvinger F Geor RJ. Adiposity, plasma insulin, leptin, lipids, and oxidative stress in mature light breed horses. J Vet Intern Med. (2013) 27:576–82. 10.1111/jvim.12056 23517373

[40] Ley RE Turnbaugh PJ Klein S Gordon JI. Human gut microbes associated with obesity. Nature (2006) 444:1022–3. 10.1038/4441022a 17183309

[41] Turnbaugh PJ Ley RE Mahowald MA Magrini V Mardis ER Gordon JI. An obesity-associated gut microbiome with increased capacity for energy harvest. Nature (2006) 444:1027–131. 10.1038/nature05414 17183312

[42] Turnbaugh PJ Backhed F Fulton L Gordon JI. Diet-induced obesity is linked to marked but reversible alterations in the mouse distal gut microbiome. Cell Host Microbe (2008) 3:213–23. 10.1016/j.chom.2008.02.015 18407065 PMC3687783

[43] Shepherd ML Ponder MA Burk AO Milton SC Swecker WS. Fibre digestibility, abundance of faecal bacteria and plasma acetate concentrations in overweight adult mares. J Nutr Sci. (2014) 3:1–11. 10.1017/jns.2014.8 25191602 PMC4153333

[44] Cartmill JA Thompson DL Storer WA Gentry LR Huff NK. Endocrine responses in mares and geldings with high body condition scores grouped by high vs. low resting leptin concentrations 1. J Anim Sci. (2003) 81:2311–21. 10.2527/2003.8192311x 12968707

[45] Kearns CF McKeever KH Roegner V Brady SM Malinowski K. Adiponectin and leptin are related to fat mass in horses. Vet J. (2005) 172:460–5. 10.1016/j.tvjl.2005.05.002 15996495

[46] Pratt-Phillips SE Owens KM Dowler LE Cloninger MT Assessment of resting insulin and leptin concentrations and their association with managerial and innate factors in horses. J Equine Vet Sci. (2010) 30:127–33. 10.1016/j.jevs.2010.01.060

[47] Shepherd ML Pleasant RS Crisman MV Werre SR Milton SC Swecker WS Jr Effects of high and moderate non-structural carbohydrate hay on insulin, glucose, triglyceride, and leptin concentrations in overweight Arabian geldings: hay non-structural carbohydrates in overweight horses. J Anim Physiol Anim Nutr. (2012b) 96:428–35. 10.1111/j.1439-0396.2011.01159.x 21575079

[48] Braid MD Daniels LM Kitts CL. Removal of PCR inhibitors from soil DNA by chemical flocculation. J Microbiol Methods (2003) 52:389–93. 10.1016/S0167-7012(02)00210-5 12531508

[49] DeAngelis KM Silver WL Thompson AW Firestone MK. Microbial communities acclimate to recurring changes in soil redox potential status. Environ Microbiol. (2010) 12:3137–49. 10.1111/j.1462-2920.2010.02286.x 20629704

[50] Griffiths RI Whiteley AS O'Donnell AG Bailey MJ. Rapid method for coextraction of DNA and RNA from natural environments for analysis of ribosomal DNA-and rRNA-based microbial community composition. Appl Environ Microbiol. (2000) 66:5488–91. 10.1128/AEM.66.12.5488-5491.2000 11097934 PMC92488

[51] Caporaso JG Lauber CL Walters WA Berg-Lyons D Huntley J Fierer N. Ultra-high-throughput microbial community analysis on the Illumina HiSeq and MiSeq platforms. ISME J. (2012) 6:1621–4. 10.1038/ismej.2012.8 22402401 PMC3400413

[52] Magoc T Salzberg SL. FLASH: fast length adjustment of short reads to improve genome assemblies. Bioinformatics (2011) 27:2957–63. 10.1093/bioinformatics/btr507 21903629 PMC3198573

[53] Caporaso JG Kuczynski J Stombaugh J Bittinger K Bushman FD Costello EK. QIIME allows analysis of high-throughput community sequencing data. Nat Methods (2010) 7:335–6. 10.1038/nmeth.f.303 20383131 PMC3156573

[54] Biddle AS Black SJ Blanchard JL. An *in vitro* Model of the horse gut microbiome enables identification of lactate-utilizing bacteria that differentially respond to starch induction. PLoS ONE (2013) 8:e77599. 10.1371/journal.pone.0077599 24098591 PMC3788102

[55] R Core Team R: A Language and Environment for Statistical Computing. Vienna: R Foundation for Statistical Computing (2012).

[56] Love MI Huber W Anders S. Moderated estimation of fold change and dispersion for RNA-Seq data with DESeq2. Genome Biol. (2014) 15:550. 10.1186/s13059-014-0550-8 25516281 PMC4302049

[57] Buff PR Dodds AC Morrison CD Whitley NC McFadin EL Daniel JA. Leptin in horses: tissue localization and relationship between peripheral concentrations of leptin and body condition. J Anim Sci. (2002) 80:2942–8. 10.2527/2002.80112942x 12462262

[58] Menni C Jackson MA Pallister T Steves CJ Spector TD Valdes AM. Gut microbiome diversity and high-fibre intake are related to lower long-term weight gain. Int J Obes. (2017) 41:1099–105. 10.1038/ijo.2017.66 28286339 PMC5500185

[59] Divers TJ Endocrine testing in horses: metabolic Syndrome and Cushing's Disease. J Equine Vet Sci. (2008) 28:315–6. 10.1016/j.jevs.2008.04.004

[60] Frank N Geor RJ Bailey SR Durham AE Johnson PJ. Equine metabolic syndrome. J Vet Intern Med. (2010) 24:467–75. 10.1111/j.1939-1676.2010.0503.x 20384947

[61] Fu J Bonder MJ Cenit MC Tigchelaar EF Maatman A Dekens JA. The Gut microbiome contributes to a substantial proportion of the variation in blood lipids. Circ Res. (2015) 117:817–24. 10.1161/CIRCRESAHA.115.306807 26358192 PMC4596485

[62] Duvallet C Gibbons SM Gurry T Irizarry RA Alm EJ. Meta-analysis of gut microbiome studies identifies disease-specific and shared responses. Nat. Commun. (2017) 8:1784. 10.1038/s41467-017-01973-8 29209090 PMC5716994

[63] Pu S Khazanehei H Jones PJ Khafipour E. Interactions between obesity status and dietary intake of monounsaturated and polyunsaturated oils on human gut microbiome profiles in the Canola Oil Multicenter Intervention Trial (COMIT). Front Microbiol. (2016) 7:1612. 10.3389/fmicb,.2016.01612 27777570 PMC5056191

[64] Yun Y Kim H-N Kim SE Heo SG Chang Y Ryu S. Comparative analysis of gut microbiota associated with body mass index in a large Korean cohort. BMC Microbiol. (2017) 17:151. 10.1186/s12866-017-1052-0 28676106 PMC5497371

[65] Turnbaugh PJ Hamady M Yatsunenko T Cantarel BL Duncan A Ley RE. A core gut microbiome in obese and lean twins. Nature (2008) 457:480–4. 10.1038/nature07540 19043404 PMC2677729

[66] Lin H An Y Hao F Wang Y Tang H. Correlations of fecal metabonomic and microbiomic changes induced by high-fat diet in the pre-obesity state. Sci Rep. (2016) 6:21618. 10.1038/srep21618 26916743 PMC4768318

[67] Xiao S Fei N Pang X Shen J Wang L Zhang B. A gut microbiota-targeted dietary intervention for amelioration of chronic inflammation underlying metabolic syndrome. FEMS Microbiol Ecol. (2014) 87:357–67. 10.1111/1574-6941.12228 24117923 PMC4255291

[68] Zhang C Zhang M Wang S Han R Cao Y Hua W. Interactions between gut microbiota, host genetics and diet relevant to development of metabolic syndromes in mice. ISME J. (2009) 4:232–41. 10.1038/ismej.2009.112 19865183

