# Supplementary material for: Microbiome and Blood Analyte Differences Point to Community and Metabolic Signatures in Lean and Obese Horses
Source: Front Vet Sci. 2018 Sep 20;5:225. doi: 10.3389/fvets.2018.00225 (PMC6158370; doi:10.3389/fvets.2018.00225)
Supplement: Supplementary file 1 [file Data_Sheet_1.PDF]

Supplementary Tables

Table S1: Demographics of horses included in this study

DNA extraction method: phenol-chloroform (PC), MoBio (MB). Farm: University of Illinois (UI), University of Massachusetts (UM), Private Owner (PO state), VA-MD Regional College of Veterinary Medicine (VM). BCS: (1-9) according to the Hennecke scale. BCS cat: 1-5.5 (Lean), 6-6.5 (Normal), 7+ (Obese). Feed: Pasture (P), Hay (H), Hay/Concentrate (HC). Age: 10 yo or less (Y), and over 10 yo (M).

Sample #	DNA extraction method	Owner	Age	Age cat	Gender	Breed	BCS	BCS cat	Feed	Housing Type
2	PC	UI	9	Y	Mare	Draft/X	8	Obese	P	Run-in
3	PC	UI	14	M	Mare	Appaloosa	7.5	Obese	P	Run-in
4	PC	UI	12	M	Mare	Appaloosa	7.5	Obese	P	Run-in
5	PC	UI	6	Y	Stallion	Quarter Horse	4.5	Lean	P	Run-in
6	PC	UI	10	Y	Stallion	Standardbred	7.5	Obese	P	Run-in
7	PC	UI	8	Y	Stallion	Standardbred	7.5	Obese	P	Run-in
8	PC	UM	8	Y	Gelding	Morgan	7.5	Obese	H	Run-in
9	PC	UM	6	Y	Mare	Morgan	7.5	Obese	H	Run-in
10	PC	UM	15	M	Gelding	Haflinger	7.5	Obese	H	Run-in
11	PC	UM	17	M	Mare	Morgan	7.5	Obese	H	Run-in
12	PC	UM	12	M	Mare	Morgan	7.5	Obese	H	Box stall
13	PC	UM	12	M	Gelding	Quarter Horse	7	Obese	H	Box stall
14	PC	UM	5	Y	Gelding	Morgan	7	Obese	H	Run-in
15	PC	UM	11	M	Gelding	Morgan	7.5	Obese	H	Run-in
16	PC	UM	5	Y	Gelding	Morgan	7	Obese	H	Run-in
17	PC	PO NH	14	M	Gelding	Icelandic	6.5	Normal	HC	Run-in
18	PC	UM	16	M	Gelding	Morgan/Thoroughbred	7	Obese	H	Box stall
19	PC	UM	12	M	Gelding	Draft	7	Obese	H	Box stall
20	PC	UM	15	M	Gelding	Morgan	7.5	Obese	H	Run-in
21	PC	PO NH	16	M	Mare	Icelandic	8	Obese	HC	Run-in
22	PC	PO NH	17	M	Gelding	Thoroughbred/X	5	Lean	HC	Box stall
23	PC	PO NH	16	M	Gelding	Thoroughbred/X	5.5	Lean	HC	Box stall
24	PC	PO NH	13	M	Gelding	Paint/X	6	Normal	HC	Box stall
25	PC	UM	7	Y	Mare	Morgan	7.5	Obese	H	Run-in
26	PC	PO NH	11	M	Mare	Paint	6.5	Normal	HC	Box stall
27	PC	PO NH	17	M	Gelding	Morgan/X	7	Obese	HC	Box stall
28	PC	UM	16	M	Gelding	Morgan/Thoroughbred	7	Obese	H	Run-in
29	PC	PO NH	10	Y	Gelding	Quarter Horse/X	6	Normal	HC	Box stall

30	PC	PO NH	12	M	Mare	Thoroughbred	6.5	Normal	HC	Box stall
31	PC	PO NH	20	M	Gelding	WB	6	Normal	HC	Box stall
32	PC	PO NH	16	M	Gelding	Hack/Shet	7	Obese	HC	Run-in
33	PC	PO NH	17	M	Gelding	Thoroughbred	6	Normal	HC	Box stall
35	PC	PO NH	12	M	Mare	Paint	6.5	Normal	HC	Run-in
36	PC	PO NH	12	M	Gelding	Quarter Horse	6	Normal	HC	Run-in
37	PC	PO NH	13	M	Mare	Quarter Horse	6	Normal	HC	Run-in
39	PC	VM	18	M	Mare	Standardbred	4	Lean	P	Run-in
40	PC	VM	20	M	Gelding	Arab	7	Obese	P	Run-in
41	PC	VM	15	M	Gelding	Quarter Horse	9	Obese	P	Run-in
42	PC	VM	11	M	Mare	Quarter Horse	8	Obese	P	Run-in
43	PC	VM	12	M	Mare	Paint	7	Obese	P	Run-in
44	PC	VM	17	M	Gelding	Thoroughbred	5	Lean	P	Run-in
46	PC	VM	20	M	Gelding	Arab	7	Obese	P	Run-in
47	PC	VM	12	M	Gelding	Hanoverian	7	Obese	P	Run-in
48	PC	VM	19	M	Mare	Thoroughbred	4	Lean	P	Run-in
49	PC	VM	18	M	Mare	Thoroughbred	4	Lean	P	Run-in
50	PC	VM	12	M	Mare	Quarter Horse	5	Lean	P	Run-in
51	PC	VM	17	M	Mare	Thoroughbred	4	Lean	P	Run-in
52	PC	VM	17	M	Mare	Thoroughbred	3	Lean	P	Run-in
53	PC	VM	14	M	Mare	Standardbred	5	Lean	P	Run-in
54	PC	VM	19	M	Mare	Standardbred	5	Lean	P	Run-in
56	PC	VM	13	M	Mare	Thoroughbred/Arab	5	Lean	P	Run-in
57	PC	VM	9	Y	Mare	Quarter Horse	7	Obese	P	Run-in
58	PC	VM	9	Y	Mare	Quarter Horse	8	Obese	P	Run-in
59	PC	VM	20	M	Gelding	Arab	7	Obese	P	Run-in
62	MB	PO DE	4	Y	Gelding	Standardbred	4	Lean	HC	Run-in
64	MB	PO DE	8	Y	Mare	Standardbred	4	Lean	HC	Run-in
67	MB	PO DE	4	Y	Gelding	Standardbred	5	Lean	HC	Run-in
69	MB	PO DE	6	Y	Mare	Standardbred	4	Lean	HC	Run-in
70	MB	PO DE	8	Y	Gelding	Standardbred	6	Normal	HC	Run-in
71	MB	PO DE	4	Y	Mare	Standardbred	5	Lean	HC	Run-in
74	PC	VM	11	M	Mare	Warmblood/X	8	Obese	P	Run-in
77	MB	PO DE	8	Y	Gelding	Thoroughbred	4.5	Lean	HC	Run-in
78	PC	PO VA	7	Y	Gelding	TN Walking Horse	8	Obese	P	Run-in
80	MB	PO DE	6	Y	Mare	Paint	6	Normal	HC	Run-in
82	MB	PO DE	8	Y	Mare	Thoroughbred	5	Lean	HC	Run-in
83	MB	PO DE	17	M	Mare	Thoroughbred	6	Normal	HC	Run-in

85	PC	VM	10	Y	Mare	Thoroughbred/X	8	Obese	P	Run-in
86	MB	PO DE	3	Y	Mare	Standardbred	5	Lean	HC	Run-in
87	MB	PO DE	12	M	Mare	Draft	6	Normal	HC	Run-in
88	MB	PO DE	2	Y	Mare	Standardbred	4	Lean	HC	Run-in
90	MB	PO DE	3	Y	Mare	Clyde/Standardbred	6.5	Normal	HC	Run-in
91	MB	PO DE	9	Y	Mare	Draft/X	8	Obese	H	Run-in
94	MB	PO DE	4	Y	Mare	Standardbred	5	Lean	HC	Run-in
96	PC	VM	13	M	Mare	Hanoverian	8	Obese	P	Run-in
98	PC	VM	16	M	Mare	Thoroughbred	5	Lean	P	Run-in
99	PC	VM	12	M	Gelding	Thoroughbred/X	7	Obese	P	Run-in
108	MB	PO DE	3	Y	Mare	Draft	6	Normal	HC	Run-in
119	MB	PO DE	13	M	Mare	Hackney/X	6	Normal	HC	Run-in

Table S2: All taxa identified and the total relative abundance in the obese, normal, and lean horse samples. Two-letter codes designations used in the network analysis and total relative abundance are shown.

Taxon lineage	Code	Relative Abundance (%)
Bacteria; Bacteroidetes; Bacteroidia; Bacteroidales;f__;g__	QE	16.244410
Bacteria; Firmicutes; Clostridia; Clostridiales;f__Ruminococcaceae;g__	LK	15.893878
Bacteria; Firmicutes; Clostridia; Clostridiales;f__Lachnospiraceae;g__	OJ	14.452446
Bacteria; Firmicutes; Clostridia; Clostridiales;f__;g__	QI	11.473584
Unassigned;Other;Other;Other;Other;Other	AA	3.780830
Bacteria; Firmicutes; Clostridia; Clostridiales;f__Ruminococcaceae;g__Ruminococcus	OK	3.094827
Bacteria; Firmicutes; Clostridia; Clostridiales;f__Mogibacteriaceae;g__	BL	2.678472
Bacteria; Spirochaetes; Spirochaetes; Spirochaetales;f__Spirochaetaceae;g__Treponema	FR	2.670410
Bacteria; Bacteroidetes; Bacteroidia; Bacteroidales;f__Prevotellaceae;g__Prevotella	DF	2.435187
Bacteria; Fibrobacteres; Fibrobacteria; Fibrobacterales;f__Fibrobacteraceae;g__Fibrobacter	EH	2.356017
Bacteria; Bacteroidetes; Bacteroidia; Bacteroidales;f__Paraprevotellaceae;g__CF231	KF	1.715601
Bacteria; Bacteroidetes; Bacteroidia; Bacteroidales;f__Paraprevotellaceae;g__YRC22	LF	1.563672
Bacteria; Firmicutes; Clostridia; Clostridiales;f__Lachnospiraceae;Other	NJ	1.352235
Bacteria; Firmicutes; Clostridia; Clostridiales;f__Veillonellaceae;g__Phascolarctobacterium	UK	1.251141
Bacteria; Bacteroidetes; Bacteroidia; Bacteroidales;f__Paraprevotellaceae;g__	JF	1.212778
Bacteria; Bacteroidetes; Bacteroidia; Bacteroidales;f__RF16;g__	EF	1.168953
Bacteria; Actinobacteria; Coriobacteriia; Coriobacteriales;f__Coriobacteriaceae;g__	YD	1.071085
Bacteria; Firmicutes; Clostridia; Clostridiales;f__Lachnospiraceae;g__Coprococcus	SJ	1.030847
Bacteria; Firmicutes; Clostridia; Clostridiales;f__Clostridiaceae;g__Clostridium	ZI	1.009161
Bacteria; Bacteroidetes; Bacteroidia; Bacteroidales;f__Bacteroidaceae;g__BF311	UE	0.882039
Bacteria; Firmicutes; Bacilli; Lactobacillales;f__Streptococcaceae;g__Streptococcus	MI	0.845326
Bacteria; Firmicutes; Clostridia; Clostridiales;f__Lachnospiraceae;g__Blautia	QJ	0.732944
Bacteria; Firmicutes; Clostridia; Clostridiales;f__Lachnospiraceae;g__Pseudobutyrivibrio	YJ	0.666919
Bacteria; Firmicutes; Clostridia; Clostridiales;f__Clostridiaceae;g__	VI	0.659299

Bacteria; Bacteroidetes; Bacteroidia; Bacteroidales;f__BS11;g__	RE	0.642893
Bacteria; Bacteroidetes; Bacteroidia; Bacteroidales;f__Paraprevotellaceae;g__Prevotella	MF	0.582616
Bacteria; Firmicutes; Clostridia; Clostridiales;f__Ruminococcaceae;g__Oscillospira	NK	0.550106
Bacteria; Actinobacteria; Coriobacteriia; Coriobacteriales;f__Coriobacteriaceae;g__Adlercreutzia	ZD	0.497567
Bacteria; Firmicutes; Clostridia; Clostridiales;f__Christensenellaceae;g__	TI	0.466590
Bacteria; Bacteroidetes; Bacteroidia; Bacteroidales;f__Porphyromonadaceae;g__Paludibacter	XE	0.443382
Bacteria; Proteobacteria; Alphaproteobacteria; f__;g__	MM	0.410118
Bacteria; Cyanobacteria; 4C0d-2; YS2;f__;g__	NG	0.404619
Bacteria; Tenericutes; Mollicutes; RF39;f__;g__	CS	0.361142
Bacteria; Firmicutes; Clostridia; Clostridiales;f__Mogibacteriaceae;g__Mogibacterium	DL	0.351654
Bacteria; Firmicutes; Erysipelotrichi; Erysipelotrichales;f__Erysipelotrichaceae;g__RFN20	WL	0.324342
Bacteria; Firmicutes; Clostridia;Clostridiales;f__Eubacteriaceae;g__Pseudoramibacter_Eubacterium	JJ	0.306375
Bacteria; Firmicutes; Clostridia; Clostridiales;Other;Other	PI	0.260408
Bacteria; Firmicutes; Erysipelotrichi; Erysipelotrichales;f__Erysipelotrichaceae;g__	PL	0.255276
Bacteria; Firmicutes; Clostridia; Clostridiales;f__Veillonellaceae;g__	RK	0.223319
Bacteria; Bacteroidetes; Bacteroidia; Bacteroidales;f__S24-7;g__	HF	0.217790
Bacteria; Firmicutes; Clostridia; Clostridiales;f__Lachnospiraceae;g__Dorea	TJ	0.214578
Bacteria; Firmicutes; Bacilli; Lactobacillales;f__Lactobacillaceae;g__Lactobacillus	KI	0.210029
Bacteria; Firmicutes; Clostridia; Clostridiales;f__Lachnospiraceae;g__Epulopiscium	UJ	0.202495
Bacteria; Firmicutes; Clostridia; Clostridiales;f__Clostridiaceae;Other	UI	0.190916
Bacteria; Firmicutes; Erysipelotrichi; Erysipelotrichales;f__Erysipelotrichaceae;g__p-75-a5	BM	0.185225
Bacteria; Spirochaetes; Spirochaetes; Sphaerochaetales;f__Sphaerochaetaceae;g__Sphaerochaeta	DR	0.170744
Bacteria; Firmicutes; Erysipelotrichi; Erysipelotrichales;f__Erysipelotrichaceae;g__Eubacterium	YL	0.160846
Bacteria; Firmicutes; Clostridia; Clostridiales;f__Lachnospiraceae;g__Ruminococcus	BK	0.157950
Bacteria; Bacteroidetes; Bacteroidia; Bacteroidales;f__Bacteroidaceae;g__Bacteroides	VE	0.122171
Bacteria; Firmicutes; Clostridia; Clostridiales;f__Lachnospiraceae;g__Roseburia	ZJ	0.119148
Bacteria; Tenericutes; Mollicutes; Anaeroplasmatales;f__Anaeroplasmataceae;g__Anaeroplasma	AS	0.106584
Bacteria; Proteobacteria; Deltaproteobacteria; GMD14H09;f__;g__	SP	0.086231
Bacteria; Bacteroidetes; Bacteroidia; Bacteroidales;f__p-2534-18B5;g__	NF	0.068896
Bacteria; Proteobacteria; Alphaproteobacteria; RF32;f__;g__	SM	0.068584
Bacteria; WPS-2;f__;g__	LS	0.067159
Bacteria; Spirochaetes; MVP-15; PL-11B10;f__;g__	CR	0.065488
Bacteria; Firmicutes; Clostridia; Clostridiales;f__Mogibacteriaceae;g__Anaerovorax	CL	0.059855
Bacteria; Firmicutes; Erysipelotrichi; Erysipelotrichales;f__Erysipelotrichaceae;g__L7A_E11	UL	0.051344
Bacteria; Firmicutes; Clostridia; Clostridiales;f__Ruminococcaceae;g__Faecalibacterium	MK	0.039903
Bacteria; Firmicutes; Clostridia; Clostridiales;f__Ruminococcaceae;Other	KK	0.037689
Bacteria; Firmicutes; Clostridia; Clostridiales;f__Eubacteriaceae;g__Anaerofustis	IJ	0.037477
Bacteria; Chloroflexi; Anaerolineae; Anaerolineales;f__Anaerolinaceae;g__SHD-231	AG	0.033823
Bacteria; Firmicutes; Clostridia; Clostridiales;f__Veillonellaceae;g__Anaerovibrio	SK	0.032552
Bacteria; Proteobacteria; Betaproteobacteria; Burkholderiales;f__Alcaligenaceae;g__Sutterella	WO	0.031322
Bacteria; Verrucomicrobia; Verruco-5; WCHB1-41;f__RFP12;g__	HS	0.030305

Bacteria; Actinobacteria; Actinobacteria; Actinomycetales;f__Micrococcaceae;g__	IC	0.028540
Bacteria; Firmicutes; Clostridia; Clostridiales;f__Dehalobacteriaceae;g__Dehalobacterium	DJ	0.027516
Bacteria; Firmicutes; Clostridia; Clostridiales;f__Peptococcaceae;g__	CK	0.027158
Bacteria; Proteobacteria; Alphaproteobacteria; Rickettsiales;f__;g__	HO	0.026801
Bacteria; Firmicutes; Clostridia; Clostridiales;f__Lachnospiraceae;g__Anaerostipes	PJ	0.025191
Bacteria; Tenericutes; Mollicutes; Anaeroplasmatales;f__Anaeroplasmataceae;g__	ZR	0.023869
Bacteria; Elusimicrobia; Endomicrobia; f__;g__	DH	0.022083
Bacteria; Firmicutes; Clostridia; Clostridiales;f__Dehalobacteriaceae;g__	CJ	0.022041
Bacteria; Tenericutes; Mollicutes; Mycoplasmatales;f__Mycoplasmataceae;g__	BS	0.020477
Bacteria; Synergistetes; Synergistia; Synergistales;f__Dethiosulfovibrionaceae;g__Pyramidobacter	JR	0.019591
Bacteria; TM7; TM7-3; CW040;f__F16;g__	RR	0.018627
Bacteria; Lentisphaerae; Lentisphaeria; Z20;f__R4-45B;g__	FM	0.018561
Bacteria; Tenericutes; RF3; ML615J-28;f__;g__	DS	0.018494
Bacteria; Firmicutes; Bacilli; Bacillales;f__Bacillaceae;g__Bacillus	JH	0.018227
Bacteria; Firmicutes; Erysipelotrichi; Erysipelotrichales;f__Erysipelotrichaceae;g__PSB-M-3	VL	0.017007
Bacteria; Tenericutes; Mollicutes ;f__;g__	VR	0.016614
Bacteria; Firmicutes; Clostridia; Clostridiales;f__Lachnospiraceae;g__Lachnospira	WJ	0.014694
Bacteria; Proteobacteria; Deltaproteobacteria; PB19;f__;g__	ZP	0.014442
Bacteria; Proteobacteria; Betaproteobacteria; Burkholderiales;f__Oxalobacteraceae;g__Oxalobacter	IP	0.013819
Bacteria; Elusimicrobia; Elusimicrobia; Elusimicrobiales;f__Elusimicrobiaceae;g__Elusimicrobium	CH	0.013726
Bacteria; Firmicutes; Erysipelotrichi; Erysipelotrichales;f__Erysipelotrichaceae;Other	OL	0.013617
Bacteria; Elusimicrobia; Elusimicrobia; Elusimicrobiales;f__Elusimicrobiaceae;g__	BH	0.012880
Bacteria; Actinobacteria; Actinobacteria; Actinomycetales;f__Micrococcaceae;g__Arthrobacter	JC	0.012859
Bacteria; Actinobacteria; Actinobacteria; Actinomycetales;f__Microbacteriaceae;g__Microbacterium	CC	0.012697
Bacteria; Actinobacteria; Actinobacteria; Actinomycetales;f__Nocardioidaceae;g__Aeromicrobium	WC	0.012646
Bacteria; Firmicutes; Clostridia; Clostridiales;f__EtOH8;g__	EJ	0.012583
Bacteria; Firmicutes; Clostridia; f__;g__	OI	0.012557
Bacteria; Lentisphaerae; Lentisphaeria; Victivallales;f__Victivallaceae;g__	EM	0.012348
Bacteria; Bacteroidetes; Bacteroidia; Bacteroidales;f__Rikenellaceae;g__	FF	0.011992
Bacteria; Actinobacteria; Actinobacteria; Actinomycetales;f__Nocardioidaceae;g__	VC	0.011414
Bacteria; Cyanobacteria; Chloroplast; Streptophyta;f__;g__	TG	0.010928
Bacteria; Spirochaetes; Spirochaetes; Spirochaetales;f__Spirochaetaceae;g__	ER	0.010919
Bacteria; Actinobacteria; Actinobacteria; Actinomycetales;f__Nocardiaceae;g__Rhodococcus	TC	0.010697
Bacteria; Firmicutes; Clostridia; Clostridiales;f__Mogibacteriaceae;Other	AL	0.009732
Bacteria; Firmicutes; Clostridia; Clostridiales;f__Lachnospiraceae;g__Butyrivibrio	RJ	0.009353
Bacteria; Firmicutes; Clostridia; Clostridiales;f__Lachnospiraceae;g__Moryella	XJ	0.009193
Bacteria; Bacteroidetes; Bacteroidia; Bacteroidales;f__Rikenellaceae;g__PW3	GF	0.008527
Bacteria; Synergistetes; Synergistia; Synergistales;f__Synergistaceae;g__	LR	0.008161
Bacteria; Actinobacteria; OPB41; f__;g__	FE	0.007458
Bacteria; Firmicutes; Clostridia; Clostridiales;f__Syntrophomonadaceae;g__Syntrophomonas	PK	0.006968
Bacteria; Actinobacteria; Coriobacteriia; Coriobacteriales;f__Coriobacteriaceae;g__Collinsella	BE	0.006736

Bacteria; Verrucomicrobia; Verrucomicrobiae; Verrucomicrobiales;f__Verrucomicrobiaceae;g__Akkermansia	JS	0.006669
Bacteria; Firmicutes; Clostridia; Clostridiales;f__Clostridiaceae;g__SMB53	AJ	0.006660
Bacteria; Bacteroidetes; Bacteroidia; Bacteroidales;f__Paraprevotellaceae;Other	IF	0.006510
Bacteria; Actinobacteria; Coriobacteriia; Coriobacteriales;f__Coriobacteriaceae;Other	XD	0.005822
Bacteria; Firmicutes; Clostridia; Clostridiales;f__Peptostreptococcaceae;g__	HK	0.005733
Bacteria; Proteobacteria; Gammaproteobacteria; Aeromonadales;f__Succinivibrionaceae;g__Ruminobacter	FQ	0.005693
Bacteria; Proteobacteria; Alphaproteobacteria; Rhizobiales;f__Rhizobiaceae;g__Agrobacterium	RN	0.005595
Bacteria; Firmicutes; Bacilli; Lactobacillales;f__Leuconostocaceae;g__Weissella	LI	0.005425
Bacteria; Firmicutes; Clostridia; Clostridiales;f__Peptococcaceae;g__Desulfurispora	FK	0.005143
Bacteria; Firmicutes; Erysipelotrichi; Erysipelotrichales;f__Erysipelotrichaceae;g__Coprobacillus	SL	0.004901
Bacteria; Firmicutes; Clostridia; Clostridiales;f__Clostridiaceae;g__Sarcina	BJ	0.004636
Bacteria; Firmicutes; Erysipelotrichi; Erysipelotrichales;f__Erysipelotrichaceae;g__cc_115	ZL	0.004570
Bacteria; Bacteroidetes; Bacteroidia; Bacteroidales;f__Prevotellaceae;g__	CF	0.004549
Bacteria; Bacteroidetes; Bacteroidia; Bacteroidales;Other;Other	PE	0.004542
Bacteria; Actinobacteria; Actinobacteria; Actinomycetales;f__Microbacteriaceae;g__	XB	0.004330
Bacteria; Proteobacteria; Betaproteobacteria; Burkholderiales;f__;g__	SO	0.004200
Bacteria; Actinobacteria; Coriobacteriia; Coriobacteriales;f__Coriobacteriaceae;g__Slackia	DE	0.004060
Bacteria; Firmicutes; Erysipelotrichi; Erysipelotrichales;f__Erysipelotrichaceae;g__gut	AM	0.004008
Bacteria; Proteobacteria; Alphaproteobacteria; Sphingomonadales;f__Sphingomonadaceae;g__Sphingomonas	PO	0.003978
Bacteria; Firmicutes; Clostridia; Clostridiales;f__Peptococcaceae;g__rc4-4	GK	0.003940
Bacteria; Firmicutes; Clostridia; Clostridiales;f__Lachnospiraceae;g__Shuttleworthia	AK	0.003860
Bacteria; Firmicutes; Clostridia; Clostridiales;f__Veillonellaceae;Other	QK	0.003599
Bacteria; Actinobacteria; Actinobacteria; Actinomycetales;f__Microbacteriaceae;Other	WB	0.003571
Bacteria; Synergistetes; Synergistia; Synergistales;f__Dethiosulfovibrionaceae;g__TG5	KR	0.003558
Bacteria; Proteobacteria; Alphaproteobacteria; Rhizobiales;f__Methylobacteriaceae;g__Methylobacterium	IN	0.003419
Bacteria; Planctomycetes; vadinHA49; PeHg47;f__;g__	LM	0.003311
Bacteria; Proteobacteria; Alphaproteobacteria; Rhizobiales;f__Hyphomicrobiaceae;g__Hyphomicrobium	EN	0.003290
Bacteria; Proteobacteria; Alphaproteobacteria; Rhizobiales;f__Hyphomicrobiaceae;g__Devosia	DN	0.003258
Bacteria; Actinobacteria; Actinobacteria; Actinomycetales;f__Williamsiaceae;g__Williamsia	WD	0.003229
Bacteria; Tenericutes; Mollicutes; Acholeplasmatales;f__;g__	XR	0.003227
Bacteria; Proteobacteria; Alphaproteobacteria; Rhizobiales;f__Hyphomicrobiaceae;g__Rhodoplanes	GN	0.003199
Bacteria; Chloroflexi; Dehalococcoidetes; Dehalococcoidales;f__Dehalococcoidaceae;g__	FG	0.003117
Bacteria; Tenericutes; Mollicutes; Acholeplasmatales;Other;Other	WR	0.002827
Bacteria; Cyanobacteria; Chloroplast; Stramenopiles;f__;g__	SG	0.002722
Bacteria; Firmicutes; Clostridia; Clostridiales;f__Lachnospiraceae;g__Lachnobacterium	VJ	0.002571
Bacteria; Firmicutes; Clostridia; Clostridiales;f__Eubacteriaceae;Other	FJ	0.002530
Bacteria; Proteobacteria; Betaproteobacteria; Burkholderiales;f__Oxalobacteraceae;g__	GP	0.002468
Bacteria; Firmicutes; Clostridia; Clostridiales;f__Veillonellaceae;g__Selenomonas	WK	0.002287
Bacteria; Synergistetes; Synergistia; Synergistales;f__Synergistaceae;g__Candidatus Tammella	MR	0.002273
Bacteria; Actinobacteria; Actinobacteria; Actinomycetales;f__Streptomycetaceae;g__Streptomyces	PD	0.002093

Bacteria; Synergistetes; Synergistia; Synergistales;f__Dethiosulfovibrionaceae;Other	HR	0.002024
Bacteria; Actinobacteria; Thermoleophilia; Solirubrobacterales;f__;g__	IE	0.002023
Bacteria; Bacteroidetes; Bacteroidia; Bacteroidales;f__Porphyromonadaceae;g__Porphyromonas	ZE	0.001995
Bacteria; Firmicutes; Bacilli; Lactobacillales;Other;Other	EI	0.001955
Bacteria; Proteobacteria; Gammaproteobacteria; Aeromonadales;f__Succinivibrionaceae;g__	DQ	0.001825
Bacteria; Chloroflexi; Thermomicrobia; JG30-KF-CM45;f__;g__	MG	0.001716
Bacteria; Proteobacteria; Gammaproteobacteria; Aeromonadales;f__Succinivibrionaceae;g__Succinivibrio	GQ	0.001688
Bacteria; Proteobacteria; Deltaproteobacteria; Desulfovibrionales;f__Desulfovibrionaceae;g__Desulfovibrio	RP	0.001667
Bacteria; Synergistetes; Synergistia; Synergistales;f__Dethiosulfovibrionaceae;g__	IR	0.001578
Bacteria; Proteobacteria; Epsilonproteobacteria; Campylobacterales;f__Campylobacteraceae;g__Campylobacter	CQ	0.001518
Bacteria; Actinobacteria; Actinobacteria; Actinomycetales;f__Microbacteriaceae;g__Mycetocola	DC	0.001518
Bacteria; Firmicutes; Bacilli; Bacillales;f__Planococcaceae;g__Solibacillus	VH	0.001482
Bacteria; Proteobacteria; Alphaproteobacteria; Rhodospirillales;f__Rhodospirillaceae;g__Skermanella	GO	0.001477
Bacteria; Firmicutes; Bacilli; Bacillales;f__Planococcaceae;g__Sporosarcina	WH	0.001468
Bacteria; Proteobacteria; Alphaproteobacteria; Rhizobiales;f__Bradyrhizobiaceae;g__Bradyrhizobium	AN	0.001451
Bacteria; Proteobacteria; Gammaproteobacteria; Pseudomonadales;f__Moraxellaceae;g__Acinetobacter	RQ	0.001441
Bacteria; Proteobacteria; Alphaproteobacteria; Rhizobiales;f__Rhizobiaceae;Other	PN	0.001392
Bacteria; Actinobacteria; Actinobacteria; Actinomycetales;f__Micromonosporaceae;g__	MC	0.001372
Bacteria; Bacteroidetes; Bacteroidia; Bacteroidales;f__Prevotellaceae;Other	BF	0.001372
Bacteria; Bacteroidetes; Sphingobacteriia; Sphingobacteriales;f__Sphingobacteriaceae;g__Pedobacter	UF	0.001366
Bacteria; Proteobacteria; Betaproteobacteria; Burkholderiales;f__Oxalobacteraceae;Other	FP	0.001357
Bacteria; Firmicutes; Erysipelotrichi; Erysipelotrichales;f__Erysipelotrichaceae;g__Bulleidia	RL	0.001288
Bacteria; Synergistetes; Synergistia; Synergistales;f__Synergistaceae;g__vadinCA02	MT	0.001276
Bacteria; Proteobacteria; Deltaproteobacteria; Myxococcales;f__;g__	UP	0.001271
Bacteria; Actinobacteria; Actinobacteria; Actinomycetales;f__Pseudonocardiaceae;g__Pseudonocardia	LD	0.001261
Bacteria; Proteobacteria; Alphaproteobacteria; Rhizobiales;f__Aurantimonadaceae;g__	VM	0.001230
Bacteria; Proteobacteria; Alphaproteobacteria; Rhodospirillales;f__;g__	AO	0.001183
Bacteria; Proteobacteria; Alphaproteobacteria; Rhizobiales;f__;g__	UM	0.001180
Bacteria; Proteobacteria; Alphaproteobacteria; Caulobacterales;f__Caulobacteraceae;g__Caulobacter	PM	0.001178
Bacteria; Actinobacteria; Actinobacteria; Actinomycetales;f__Actinomycetaceae;g__	XA	0.001176
Bacteria; Proteobacteria; Betaproteobacteria; Burkholderiales;Other;Other	RO	0.001170
Bacteria; Actinobacteria; Actinobacteria; Actinomycetales;f__Corynebacteriaceae;g__Corynebacterium	JB	0.001124
Bacteria; Verrucomicrobia; Opitutae; HA64;f__;g__	ES	0.001115
Bacteria; Actinobacteria; Actinobacteria; Actinomycetales;f__Actinomycetaceae;g__Arcanobacterium	ZA	0.001086
Bacteria; Actinobacteria; Actinobacteria; Actinomycetales;f__Actinomycetaceae;Other	WA	0.001045
Bacteria;Other;Other;Other;Other;Other	EA	0.001044
Bacteria; Firmicutes; Clostridia; Clostridiales;f__Veillonellaceae;g__Succiniclasticum	XK	0.001037
Bacteria; Firmicutes; Clostridia; SHA-98;f__;g__	NL	0.001018
Bacteria; Proteobacteria; Gammaproteobacteria; Aeromonadales;f__Succinivibrionaceae;g__Anaerobiospirillum	EQ	0.001015

Bacteria; Proteobacteria; Alphaproteobacteria; Rhizobiales;f__Bradyrhizobiaceae;g__	YM	0.000999
Bacteria; Actinobacteria; Acidimicrobiia; Acidimicrobiales;f__;g__	PA	0.000969
Bacteria; Cyanobacteria; Chloroplast; Chlorophyta;f__Chlamydomonadaceae;g__	QG	0.000969
Bacteria; Actinobacteria; Acidimicrobiia; Acidimicrobiales;f__C111;g__	RA	0.000917
Bacteria; Firmicutes;Other;Other;Other;Other	FH	0.000901
Bacteria; Proteobacteria; Gammaproteobacteria; Pseudomonadales;f__Moraxellaceae;g__Psychrobacter	SQ	0.000900
Bacteria; Actinobacteria; Actinobacteria; Actinomycetales;f__;g__	VA	0.000839
Bacteria; Cyanobacteria; Chloroplast; Chlorophyta;f__Trebouxiophyceae;g__	RG	0.000838
Bacteria; Actinobacteria; Thermoleophilia; Gaiellales;f__Gaiellaceae;g__	HE	0.000826
Bacteria; Proteobacteria; Betaproteobacteria; Burkholderiales;f__Comamonadaceae;g__	ZO	0.000795
Bacteria; Proteobacteria; Alphaproteobacteria; Rhizobiales;f__Hyphomicrobiaceae;g__Pedomicrobium	FN	0.000733
Bacteria; Proteobacteria; Alphaproteobacteria; Rhodospirillales;f__Rhodospirillaceae;g__	EO	0.000712
Bacteria; Firmicutes; Clostridia; Clostridiales;f__Eubacteriaceae;g__Acetobacterium	HJ	0.000707
Bacteria; Actinobacteria; Actinobacteria; Actinomycetales;f__Nocardioidaceae;g__Nocardioides	ZC	0.000701
Bacteria; Proteobacteria; Deltaproteobacteria; Desulfovibrionales;f__Desulfovibrionaceae;g__	QP	0.000701
Bacteria; Actinobacteria; Thermoleophilia; Solirubrobacterales;f__Solirubrobacteraceae;g__	ME	0.000693
Bacteria; Cyanobacteria; Chloroplast; Chlorophyta;f__;g__	OG	0.000677
Bacteria; Firmicutes; Clostridia; Clostridiales;f__Clostridiaceae;g__02d06	WI	0.000677
Bacteria; Actinobacteria; Actinobacteria; Actinomycetales;f__Mycobacteriaceae;g__Mycobacterium	QC	0.000667
Bacteria; Actinobacteria; Actinobacteria; Actinomycetales;f__Nocardioidaceae;g__Friedmanniella	XC	0.000666
Bacteria; Proteobacteria; Alphaproteobacteria; Rhizobiales;f__Bradyrhizobiaceae;g__Balneimonas	ZM	0.000657
Bacteria; Planctomycetes; Planctomycetia; Pirellulales;f__Pirellulaceae;g__	KM	0.000641
Bacteria; Proteobacteria; Alphaproteobacteria; Rhizobiales;f__Phyllobacteriaceae;g__	MN	0.000639
Bacteria; Firmicutes; Clostridia; Clostridiales;f__Tissierellaceae;g__Peptoniphilus	KL	0.000636
Bacteria; Firmicutes; Bacilli; Bacillales;f__Paenibacillaceae;g__Paenibacillus	OH	0.000631
Bacteria; Proteobacteria; Alphaproteobacteria; Rhizobiales;f__Phyllobacteriaceae;Other	LN	0.000625
Bacteria; Actinobacteria; Actinobacteria; Actinomycetales;f__Propionibacteriaceae;g__	FD	0.000622
Bacteria; Firmicutes; Clostridia; Clostridiales;f__Tissierellaceae;g__Finegoldia	GL	0.000618
Bacteria; Proteobacteria; Alphaproteobacteria; Caulobacterales;f__Caulobacteraceae;g__	OM	0.000614
Bacteria; Proteobacteria; Alphaproteobacteria; Rhodospirillales;f__Acetobacteraceae;g__	CO	0.000611
Bacteria; Firmicutes; Clostridia; Clostridiales;f__Veillonellaceae;g__Schwartzia	VK	0.000609
Bacteria; Actinobacteria; Actinobacteria; Actinomycetales;f__Cellulomonadaceae;Other	HB	0.000606
Bacteria; Proteobacteria; Gammaproteobacteria; Enterobacteriales;f__Enterobacteriaceae;g__	IQ	0.000583
Bacteria; Proteobacteria; Alphaproteobacteria; Rhizobiales;f__Hyphomicrobiaceae;g__	CN	0.000573
Bacteria; Acidobacteria; Acidobacteriia; Acidobacteriales;f__Koribacteraceae;g__	HA	0.000568
Bacteria; Actinobacteria; Actinobacteria; Actinomycetales;f__Gordoniaceae;g__Gordonia	PB	0.000561
Bacteria; Actinobacteria; Acidimicrobiia; Acidimicrobiales;f__EB1017;g__	SA	0.000527
Bacteria; Actinobacteria; Actinobacteria; Actinomycetales;f__Propionibacteriaceae;g__Microlunatus	GD	0.000517
Bacteria; Actinobacteria; Actinobacteria; Actinomycetales;f__Intrasporangiaceae;Other	QB	0.000506
Bacteria; Proteobacteria; Alphaproteobacteria; Rhizobiales;f__Beijerinckiaceae;g__	XM	0.000490

Bacteria; Acidobacteria; Solibacteres; Solibacterales;f__;g__	JA	0.000486
Bacteria; Proteobacteria; Alphaproteobacteria; Rhodobacterales;f__Rhodobacteraceae;g__Amaricoccus	WN	0.000484
Bacteria; Actinobacteria; Actinobacteria; Actinomycetales;f__Microbacteriaceae;g__Pseudoclavibacter	EC	0.000481
Bacteria; Firmicutes; Clostridia; Clostridiales;f__Clostridiaceae;g__Alkaliphilus	XI	0.000462
Bacteria; Actinobacteria; Thermoleophilia; Solirubrobacterales;f__Patulibacteraceae;g__	KE	0.000451
Bacteria; Chloroflexi; Ellin6529; f__;g__	GG	0.000440
Bacteria; Firmicutes; Clostridia; Clostridiales;f__Veillonellaceae;g__Veillonella	YK	0.000433
Bacteria; Actinobacteria; Actinobacteria; Actinomycetales;f__Cellulomonadaceae;g__Cellulomonas	IB	0.000409
Bacteria; Actinobacteria; Actinobacteria; Actinomycetales;f__Nakamurellaceae;g__	RC	0.000409
Bacteria; Actinobacteria; Actinobacteria; Actinomycetales;f__Microbacteriaceae;g__Curtobacterium	AC	0.000402
Bacteria; Proteobacteria; Gammaproteobacteria; Pasteurellales;f__Pasteurellaceae;g__Actinobacillus	PQ	0.000398
Bacteria; Proteobacteria; Alphaproteobacteria; Sphingomonadales;f__Sphingomonadaceae;g__Sphingobium	OO	0.000383
Bacteria; Proteobacteria; Alphaproteobacteria; Rhizobiales;f__Methylocystaceae;g__	JN	0.000376
Bacteria; Proteobacteria; Gammaproteobacteria; Xanthomonadales;f__Xanthomonadaceae;g__Luteimonas	AR	0.000373
Bacteria; Firmicutes; Clostridia; Clostridiales;f__Tissierellaceae;g__Gallicola	HL	0.000371
Bacteria; Firmicutes; Clostridia; Clostridiales;f__Tissierellaceae;g__Helcococcus	IL	0.000367
Bacteria; Actinobacteria; Actinobacteria; Actinomycetales;f__Geodermatophilaceae;g__	MB	0.000365
Bacteria; Actinobacteria; Actinobacteria; Actinomycetales;f__Pseudonocardiaceae;g__Actinomycetospora	JD	0.000364
Bacteria; Proteobacteria; Gammaproteobacteria; Xanthomonadales;f__Xanthomonadaceae;g__	YQ	0.000362
Bacteria; Proteobacteria; Alphaproteobacteria; Rhizobiales;f__Rhizobiaceae;g__Kaistia	SN	0.000359
Bacteria; Firmicutes; Erysipelotrichi; Erysipelotrichales;f__Erysipelotrichaceae;g__Asteroleplasma	QL	0.000358
Bacteria; Actinobacteria; Actinobacteria; Actinomycetales;f__Microbacteriaceae;g__Agrococcus	YB	0.000352
Bacteria; Actinobacteria; Actinobacteria; Actinomycetales;f__Microbacteriaceae;g__Salinibacterium	GC	0.000344
Bacteria; Actinobacteria; Actinobacteria; Actinomycetales;f__Actinosynnemataceae;g__Lentzea	DB	0.000341
Bacteria; Chloroflexi; Anaerolineae; SBR1031;f__oc28;g__	BG	0.000341
Bacteria; TM7; TM7-3; I025;f__;g__	TR	0.000338
Bacteria; Chloroflexi; Gitt-GS-136; f__;g__	HG	0.000338
Bacteria; Bacteroidetes; Saprospirae; Saprospirales;f__Chitinophagaceae;g__Sediminibacterium	YF	0.000332
Bacteria; Synergistetes; Synergistia; Synergistales;Other;Other	GR	0.000332
Bacteria; Fusobacteria; Fusobacteriia; Fusobacteriales;f__Fusobacteriaceae;g__Fusobacterium	CM	0.000330
Bacteria; Firmicutes; Bacilli; Bacillales;f__Paenibacillaceae;g__	KH	0.000329
Bacteria; Firmicutes; Bacilli; Bacillales;f__Planococcaceae;g__	QH	0.000311
Bacteria; Actinobacteria; Actinobacteria; Actinomycetales;f__Geodermatophilaceae;g__Geodermatophilus	NB	0.000304
Bacteria; Bacteroidetes; Flavobacteriia; Flavobacteriales;f__Weeksellaceae;g__Chryseobacterium	RF	0.000301
Bacteria; Firmicutes; Clostridia; Clostridiales;f__Tissierellaceae;g__Anaerococcus	FL	0.000299
Bacteria; Actinobacteria; MB-A2-108; 0319-7L14;f__;g__	EE	0.000292
Bacteria; Actinobacteria; Actinobacteria; Actinomycetales;f__Pseudonocardiaceae;g__Saccharopolyspora	ND	0.000292
Bacteria; Actinobacteria; Actinobacteria; Actinomycetales;f__Nocardioidaceae;g__Kribbella	YC	0.000291
Bacteria; Actinobacteria; Acidimicrobiia; Acidimicrobiales;f__Microthrixaceae;g__	TA	0.000279

Bacteria; Bacteroidetes; Bacteroidia; Bacteroidales;f__Porphyromonadaceae;g__Tannerella	AF	0.000277
Bacteria; Firmicutes; Bacilli; Bacillales;f__Planococcaceae;g__Rummeliibacillus	UH	0.000261
Bacteria; Firmicutes; Bacilli; Bacillales;f__Staphylococcaceae;g__Jeotgalicoccus	XH	0.000255
Bacteria; Firmicutes; Clostridia; Clostridiales;f__Gracilibacteraceae;g__	LJ	0.000253
Bacteria; Bacteroidetes; Bacteroidia; Bacteroidales;f__Porphyromonadaceae;g__	WE	0.000249
Bacteria; Proteobacteria; Gammaproteobacteria; Xanthomonadales;f__Sinobacteraceae;g__Steroidobacter	WQ	0.000247
Bacteria; Actinobacteria; Actinobacteria; Actinomycetales;Other;Other	UA	0.000242
Bacteria; Proteobacteria; Alphaproteobacteria; Rhodospirillales;f__Acetobacteraceae;Other	BO	0.000237
Bacteria; Firmicutes; Bacilli; Lactobacillales;f__Aerococcaceae;g__Facklamia	GI	0.000237
Bacteria; Actinobacteria; Actinobacteria; Actinomycetales;f__Microbacteriaceae;g__Agromyces	ZB	0.000229
Bacteria; Proteobacteria; Alphaproteobacteria; Rhizobiales;f__Methylobacteriaceae;g__	HN	0.000229
Bacteria; Actinobacteria; Actinobacteria; Actinomycetales;f__Kineosporiaceae;g__Kineococcus	VB	0.000228
Bacteria; Cyanobacteria; Synechococcophycideae; Pseudanabaenales;f__Pseudanabaenaceae;g__Arthronema	ZG	0.000227
Bacteria; Proteobacteria; Gammaproteobacteria; Pseudomonadales;f__Pseudomonadaceae;g__Pseudomonas	UQ	0.000221
Bacteria; Chloroflexi; Chloroflexi; Roseiflexales;f__;g__	DG	0.000219
Bacteria; Proteobacteria; Gammaproteobacteria; Xanthomonadales;f__Xanthomonadaceae;Other	XQ	0.000212
Bacteria; Bacteroidetes; Bacteroidia; Bacteroidales;f__Porphyromonadaceae;g__Parabacteroides	YE	0.000210
Bacteria; Proteobacteria; Alphaproteobacteria; Sphingomonadales;f__Sphingomonadaceae;g__Sphingopyxis	QO	0.000210
Bacteria; Proteobacteria; Betaproteobacteria; Burkholderiales;f__Alcaligenaceae;g__Achromobacter	UO	0.000206
Bacteria; Proteobacteria; Alphaproteobacteria; Sphingomonadales;f__Sphingomonadaceae;g__Novosphingobium	NO	0.000205
Bacteria; Proteobacteria; Alphaproteobacteria; Sphingomonadales;f__Sphingomonadaceae;g__	LO	0.000203
Bacteria; Proteobacteria; Betaproteobacteria; Burkholderiales;f__Comamonadaceae;g__Variovorax	EP	0.000198
Bacteria; Proteobacteria; Alphaproteobacteria; Rhizobiales;f__Rhizobiaceae;g__Rhizobium	TN	0.000197
Bacteria; Actinobacteria; Actinobacteria; Actinomycetales;f__Nocardiopsaceae;g__	BD	0.000194
Bacteria; Proteobacteria; Betaproteobacteria; Burkholderiales;f__Burkholderiaceae;g__Burkholderia	XO	0.000191
Bacteria; Proteobacteria; Alphaproteobacteria; Rhizobiales;f__Xanthobacteraceae;g__Labrys	UN	0.000189
Bacteria; TM7; SC3; f__;g__	OR	0.000188
Bacteria; Actinobacteria; Actinobacteria; Actinomycetales;f__Frankiaceae;g__	LB	0.000187
Bacteria; Firmicutes; Bacilli; Bacillales;f__Staphylococcaceae;g__Staphylococcus	YH	0.000187
Bacteria; Proteobacteria; Alphaproteobacteria; Rhodobacterales;f__Rhodobacteraceae;g__Paracoccus	XN	0.000181
Bacteria; Proteobacteria; Gammaproteobacteria; Enterobacteriales;f__Enterobacteriaceae;g__Erwinia	JQ	0.000181
Bacteria; Proteobacteria; Alphaproteobacteria; Rhodobacterales;f__Rhodobacteraceae;g__	VN	0.000179
Bacteria; Proteobacteria; Deltaproteobacteria; Myxococcales;f__Haliangiaceae;g__	WP	0.000171
Bacteria;f__;g__	FA	0.000169
Bacteria; Actinobacteria; Coriobacteriia; Coriobacteriales;f__Coriobacteriaceae;g__Eggerthella	CE	0.000167
Bacteria; Firmicutes; Erysipelotrichi; Erysipelotrichales;f__Erysipelotrichaceae;g__Sharpea	XL	0.000164
Bacteria; Actinobacteria; Actinobacteria; Actinomycetales;f__Microbacteriaceae;g__Leucobacter	BC	0.000162
Bacteria; Proteobacteria; Alphaproteobacteria; Rhizobiales;f__Brucellaceae;Other	BN	0.000157
Bacteria; Verrucomicrobia; Verruco-5; WCHB1-41;f__WCHB1-25;g__	IS	0.000154

Bacteria; Firmicutes; Clostridia; Clostridiales;f__Tissierellaceae;g__Tissierella_Soehngenia	ML	0.000154
Bacteria; Actinobacteria; Actinobacteria; Actinomycetales;f__Actinomycetaceae;g__Actinomyces	YA	0.000154
Bacteria; Actinobacteria; Actinobacteria; Actinomycetales;f__Intrasporangiaceae;g__Phycicoccus	SB	0.000153
Bacteria; Actinobacteria; Actinobacteria; Actinomycetales;f__Sanguibacteraceae;g__Sanguibacter	OD	0.000152
Bacteria; Firmicutes; Clostridia; Clostridiales;f__Peptostreptococcaceae;g__Peptostreptococcus	IK	0.000151
Bacteria; Cyanobacteria; Synechococcophycideae; Pseudanabaenales;f__Pseudanabaenaceae;g__Leptolyngbya	AH	0.000147
Archaea; Euryarchaeota; Thermoplasmata; E2;f__Methanomassiliicoccaceae;g__vadinCA11	DA	0.000146
Bacteria; Proteobacteria; Alphaproteobacteria; Sphingomonadales;f__Erythrobacteraceae;g__	JO	0.000146
Bacteria; Actinobacteria; Actinobacteria; Actinomycetales;f__Streptosporangiaceae;g__Streptosporangium	TD	0.000145
Bacteria; Acidobacteria; Acidobacteria-6; iii1-15;f__;g__	GA	0.000144
Bacteria; Actinobacteria; Actinobacteria; Actinomycetales;f__Micromonosporaceae;Other	LC	0.000143
Bacteria; Actinobacteria; Actinobacteria; Actinomycetales;f__Streptosporangiaceae;g__Sphaerisporangium	SD	0.000140
Bacteria; Firmicutes; Clostridia; Clostridiales;f__Veillonellaceae;g__Dialister	TK	0.000138
Bacteria; Bacteroidetes; Sphingobacteriia; Sphingobacteriales;f__Sphingobacteriaceae;g__	TF	0.000136
Bacteria; Proteobacteria; Betaproteobacteria; Burkholderiales;f__Comamonadaceae;Other	YO	0.000130
Bacteria; Proteobacteria; Deltaproteobacteria; Desulfobacterales;f__Desulfobulbaceae;g__Desulfobulbus	PP	0.000128
Bacteria; Proteobacteria; Gammaproteobacteria; Enterobacteriales;f__Enterobacteriaceae;Other	HQ	0.000126
Bacteria; Proteobacteria; Gammaproteobacteria; Xanthomonadales;f__Xanthomonadaceae;g__Dokdonella	ZQ	0.000125
Bacteria; Firmicutes; Bacilli; Lactobacillales;f__Aerococcaceae;g__	FI	0.000124
Bacteria; Firmicutes; Bacilli; Bacillales;f__Thermoactinomycetaceae;g__	AI	0.000123
Bacteria; Bacteroidetes; Saprospirae; Saprospirales;f__Chitinophagaceae;g__	WF	0.000120
Bacteria; Proteobacteria; Alphaproteobacteria; Rhizobiales;f__Phyllobacteriaceae;g__Mesorhizobium	ON	0.000120
Bacteria; Proteobacteria; Betaproteobacteria; Tremblayales;f__;g__	OP	0.000118
Bacteria; Firmicutes; Bacilli; Bacillales;f__Planococcaceae;g__Planomicrobium	TH	0.000116
Bacteria; Firmicutes; Bacilli; Bacillales;f__Planococcaceae;g__Lysinibacillus	SH	0.000116
Bacteria; Actinobacteria; Actinobacteria; Actinomycetales;f__Propionibacteriaceae;g__Propionibacterium	HD	0.000114
Bacteria; Proteobacteria; Gammaproteobacteria; Pasteurellales;f__Pasteurellaceae;g__	OQ	0.000113
Bacteria; TM7; TM7-1; f__;g__	PR	0.000111
Bacteria; Firmicutes; Bacilli; Bacillales;f__Planococcaceae;Other	PH	0.000111
Bacteria; Proteobacteria; Betaproteobacteria; Neisseriales;f__Neisseriaceae;g__	KP	0.000105
Bacteria; Proteobacteria; Betaproteobacteria; Burkholderiales;f__Alcaligenaceae;g__Pigmentiphaga	VO	0.000104
Bacteria; Planctomycetes; Planctomycetia; Gemmatales;f__Isosphaeraceae;g__	JM	0.000104
Bacteria; Proteobacteria; Alphaproteobacteria; Sphingomonadales;f__Sphingomonadaceae;Other	KO	0.000103
Bacteria; Proteobacteria; Alphaproteobacteria; Rhizobiales;Other;Other	TM	0.000101
Bacteria; Firmicutes; Clostridia; Clostridiales;f__Gracilibacteraceae;g__Gracilibacter	MJ	0.000101
Bacteria; Firmicutes; Bacilli; Lactobacillales;f__Enterococcaceae;g__Enterococcus	JI	0.000099
Bacteria; Actinobacteria; Actinobacteria; Actinomycetales;f__Actinopolysporaceae;g__	BB	0.000098
Bacteria; Actinobacteria; Actinobacteria; Actinomycetales;f__Thermomonosporaceae;g__Actinoallomurus	UD	0.000097
Bacteria; Proteobacteria; Alphaproteobacteria; Rhizobiales;f__Phyllobacteriaceae;g__Aminobacter	NN	0.000097

Bacteria; Actinobacteria; Actinobacteria; Actinomycetales;f__Micromonosporaceae;g__Virgisporangium	PC	0.000096
Bacteria; Acidobacteria; Solibacteres; Solibacterales;f__Solibacteraceae;g__	KA	0.000094
Bacteria; Thermi; Deinococci; Deinococcales;f__Deinococcaceae;g__Deinococcus	MS	0.000093
Bacteria; Bacteroidetes; Saprospirae; Saprospirales;f__Chitinophagaceae;g__Flavihumibacter	XF	0.000093
Bacteria; Proteobacteria; Alphaproteobacteria; Rhodobacterales;f__Rhodobacteraceae;g__Rhodobacter	YN	0.000091
Bacteria; Firmicutes; Bacilli; Bacillales;Other;Other	GH	0.000091
Bacteria; Proteobacteria; Betaproteobacteria; Nitrosomonadales;f__;g__	LP	0.000090
Bacteria; Firmicutes; Bacilli; Bacillales;f__;g__	HH	0.000089
Bacteria; Proteobacteria; Alphaproteobacteria; Rhizobiales;f__Rhizobiaceae;g__	QN	0.000089
Bacteria; Firmicutes; Bacilli; Bacillales;f__Thermoactinomycetaceae;g__Shimazuella	CI	0.000087
Bacteria; Tenericutes; Mollicutes;Other;Other;Other	UR	0.000084
Bacteria; Bacteroidetes; Bacteroidia; Bacteroidales;f__Bacteroidaceae;Other	SE	0.000083
Bacteria; Proteobacteria; Alphaproteobacteria; Caulobacterales;f__Caulobacteraceae;g__Phenylobacterium	RM	0.000083
Bacteria; Verrucomicrobia; Pedosphaerae; Pedosphaerales;f__R4-41B;g__	KS	0.000080
Bacteria; Actinobacteria; Actinobacteria; Actinomycetales;f__Micrococcaceae;Other	HC	0.000078
Bacteria; Firmicutes; Clostridia; Clostridiales;f__Peptococcaceae;g__Desulfotomaculum	EK	0.000078
Bacteria; Actinobacteria; Actinobacteria; Actinomycetales;f__Micromonosporaceae;g__Actinoplanes	NC	0.000078
Bacteria; Actinobacteria; Actinobacteria; Actinomycetales;f__Pseudonocardiaceae;g__Saccharomonospora	MD	0.000077
Bacteria; Proteobacteria; Alphaproteobacteria; Sphingomonadales;f__Sphingomonadaceae;g__Kaistobacter	MO	0.000077
Bacteria; Firmicutes; Bacilli; Lactobacillales;f__Carnobacteriaceae;g__Carnobacterium	HI	0.000077
Bacteria; Proteobacteria; Gammaproteobacteria; Legionellales;f__Coxiellaceae;g__	LQ	0.000073
Bacteria; OD1; ABY1; f__;g__	IM	0.000073
Bacteria; Bacteroidetes; Flavobacteriia; Flavobacteriales;f__Flavobacteriaceae;g__Flavobacterium	QF	0.000073
Bacteria; Firmicutes; Bacilli; Bacillales;f__Paenibacillaceae;g__Ammoniphilus	LH	0.000072
Bacteria; Proteobacteria; Deltaproteobacteria; Myxococcales;f__Polyangiaceae;g__Sorangium	YP	0.000072
Bacteria; Actinobacteria; Actinobacteria; Actinomycetales;f__Micrococcaceae;g__Kocuria	KC	0.000071
Bacteria; Proteobacteria; Betaproteobacteria; Burkholderiales;f__Comamonadaceae;g__Polaromonas	BP	0.000071
Bacteria; Bacteroidetes; Cytophagia; Cytophagales;f__Cytophagaceae;g__	PF	0.000071
Bacteria; Cyanobacteria; Chloroplast; Chlorophyta;f__Chlamydomonadaceae;Other	PG	0.000069
Bacteria; Proteobacteria; Gammaproteobacteria; Methylococcales;f__Crenotrichaceae;g__Crenothrix	NQ	0.000069
Bacteria; Firmicutes; Clostridia; Clostridiales;f__Peptostreptococcaceae;g__Tepidibacter	JK	0.000069
Bacteria; Cyanobacteria; Nostocophycideae; Nostocales;f__Nostocaceae;Other	UG	0.000068
Bacteria; Acidobacteria; Solibacteres; Solibacterales;f__Solibacteraceae;g__Candidatus Solibacter	LA	0.000066
Bacteria; Actinobacteria; Actinobacteria; Actinomycetales;f__Nocardioidaceae;g__Pimelobacter	AD	0.000066
Bacteria; Proteobacteria; Betaproteobacteria; SC-I-84;f__;g__	NP	0.000066
Bacteria; Chloroflexi; Thermomicrobia; AKYG1722;f__;g__	LG	0.000066
Bacteria; Cyanobacteria; Oscillatoriophycideae; Oscillatoriales;f__Phormidiaceae;g__Phormidium	YG	0.000066
Bacteria; Actinobacteria; Actinobacteria; Actinomycetales;f__Glycomycetaceae;g__Glycomyces	OB	0.000065
Bacteria; Actinobacteria; Actinobacteria; Actinomycetales;f__Nocardiopsaceae;g__Nocardiopsis	CD	0.000065
Bacteria; Actinobacteria; Actinobacteria; Actinomycetales;f__Nocardiopsaceae;g__Thermobifida	DD	0.000065

Bacteria; Actinobacteria; Actinobacteria; Actinomycetales;f__Thermomonosporaceae;g__Actinocorallia	VD	0.000065
Bacteria; Firmicutes; Bacilli; Bacillales;f__Thermoactinomycetaceae;g__Planifilum	BI	0.000065
Bacteria; Firmicutes; Bacilli; Bacillales;f__Thermoactinomycetaceae;Other	ZH	0.000065
Bacteria; Actinobacteria; Actinobacteria; Actinomycetales;f__Microbacteriaceae;g__Rathayibacter	FC	0.000064
Bacteria; Actinobacteria; Thermoleophilia; Gaiellales;f__;g__	GE	0.000063
Bacteria; Acidobacteria; Acidobacteriia; Acidobacteriales;f__Koribacteraceae;g__Candidatus Koribacter	IA	0.000063
Bacteria; Actinobacteria; Actinobacteria; Actinomycetales;f__Pseudonocardiaceae;g__Amycolatopsis	KD	0.000063
Bacteria; Chloroflexi; Anaerolineae; envOPS12;f__;g__	CG	0.000063
Bacteria; Chloroflexi; Chloroflexi; Roseiflexales;f__Kouleothrixaceae;g__	EG	0.000063
Bacteria; Proteobacteria; Betaproteobacteria; Burkholderiales;f__Alcaligenaceae;g__	TO	0.000062
Bacteria; Proteobacteria; Betaproteobacteria; Rhodocyclales;f__Rhodocyclaceae;g__	MP	0.000061
Bacteria; Bacteroidetes; Flavobacteriia; Flavobacteriales;f__Weeksellaceae;g__Wautersiella	SF	0.000061
Bacteria; Actinobacteria;Other;Other;Other;Other	OA	0.000059
Bacteria; Actinobacteria; Actinobacteria; Actinomycetales;f__Streptosporangiaceae;g__	QD	0.000059
Bacteria; Verrucomicrobia; Verruco-5; WCHB1-41;f__;g__	GS	0.000059
Bacteria; Proteobacteria; Alphaproteobacteria; Caulobacterales;f__Caulobacteraceae;Other	NM	0.000059
Bacteria; Proteobacteria; Gammaproteobacteria; Legionellales;f__Legionellaceae;g__Tatlockia	MQ	0.000059
Bacteria; Bacteroidetes; Sphingobacteriia; Sphingobacteriales;f__Sphingobacteriaceae;g__Sphingobacterium	VF	0.000058
Bacteria; Firmicutes; Clostridia; Clostridiales;f__Tissierellaceae;g__Sedimentibacter	LL	0.000058
Bacteria; Firmicutes; Bacilli; Bacillales;f__Paenibacillaceae;g__Cohnella	NH	0.000057
Bacteria; Firmicutes; Erysipelotrichi; Erysipelotrichales;f__Erysipelotrichaceae;g__Holdemania	TL	0.000057
Bacteria; Firmicutes; Clostridia; Clostridiales;f__Clostridiaceae;g__Caloramator	YI	0.000057
Bacteria; Firmicutes; Bacilli; Bacillales;f__Alicyclobacillaceae;g__Alicyclobacillus	IH	0.000052
Bacteria; Chloroflexi; S085; f__;g__	KG	0.000051
Bacteria; Proteobacteria; Betaproteobacteria; Burkholderiales;f__Comamonadaceae;g__Roseateles	DP	0.000051
Bacteria; Proteobacteria; Gammaproteobacteria; Pseudomonadales;f__Pseudomonadaceae;g__	TQ	0.000048
Bacteria; Firmicutes; Clostridia; Clostridiales;f__Eubacteriaceae;g__	GJ	0.000048
Bacteria; Bacteroidetes; Cytophagia; Cytophagales;f__Cyclobacteriaceae;g__	OF	0.000047
Bacteria; Actinobacteria; Actinobacteria; Actinomycetales;f__Micromonosporaceae;g__Dactylosporangium	OC	0.000046
Bacteria; Fusobacteria; Fusobacteriia; Fusobacteriales;f__Leptotrichiaceae;g__Leptotrichia	DM	0.000046
Bacteria; Firmicutes; Clostridia; Clostridiales;f__Peptococcaceae;g__Desulfosporosinus	DK	0.000046
Bacteria; Firmicutes; Clostridia; Clostridiales;f__Tissierellaceae;Other	EL	0.000045
Bacteria; Proteobacteria; Betaproteobacteria; Methylophilales;f__Methylophilaceae;g__	JP	0.000045
Bacteria; Proteobacteria; Deltaproteobacteria; Myxococcales;f__Cystobacterineae;g__	VP	0.000045
Bacteria; Proteobacteria; Deltaproteobacteria; Spirobacillales;f__;g__	AQ	0.000045
Bacteria; Actinobacteria; Actinobacteria; Actinomycetales;f__Intrasporangiaceae;g__	RB	0.000044
Bacteria; Actinobacteria; Thermoleophilia; Solirubrobacterales;f__Solirubrobacteraceae;g__Solirubrobacter	NE	0.000043
Bacteria; Actinobacteria; Actinobacteria; Actinomycetales;f__Actinomycetaceae;g__Trueperella	AB	0.000043
Bacteria; Tenericutes; Mollicutes; Acholeplasmatales;f__Acholeplasmataceae;g__Acholeplasma	YR	0.000042
Bacteria; Verrucomicrobia; Opitutae; Cerasicoccales;f__Cerasicoccaceae;g__	FS	0.000042

Bacteria; Actinobacteria; Actinobacteria; Actinomycetales;f__Kineosporiaceae;Other	TB	0.000042
Bacteria; TM7; TM7-3; I025;Other;Other	SR	0.000042
Bacteria; Actinobacteria; Actinobacteria; Actinomycetales;f__Brevibacteriaceae;g__Brevibacterium	GB	0.000042
Bacteria; Firmicutes; Clostridia; Clostridiales;f__Acidaminobacteraceae;g__	ZK	0.000042
Bacteria; Actinobacteria; Actinobacteria; Actinomycetales;f__Kineosporiaceae;g__	UB	0.000041
Bacteria; Proteobacteria; Gammaproteobacteria; Pasteurellales;f__Pasteurellaceae;g__Aggregatibacter	QQ	0.000040
Bacteria; TM7; TM7-3; f__;g__	QR	0.000040
Bacteria; Proteobacteria; Betaproteobacteria; Burkholderiales;f__Oxalobacteraceae;g__Janthinobacterium	HP	0.000037
Bacteria; Proteobacteria; Alphaproteobacteria; Rhodospirillales;f__Acetobacteraceae;g__Roseococcus	DO	0.000032
Bacteria; Actinobacteria; Actinobacteria; Actinomycetales;f__Dietziaceae;g__Dietzia	KB	0.000032
Bacteria; Firmicutes; Bacilli; Gemellales;f__Gemellaceae;g__Gemella	DI	0.000032
Bacteria; Actinobacteria; Thermoleophilia; Solirubrobacterales;f__Conexibacteraceae;g__	JE	0.000031
Bacteria; Proteobacteria; Alphaproteobacteria; Caulobacterales;f__Caulobacteraceae;g__Mycoplana	QM	0.000031
Bacteria; Cyanobacteria; Nostocophycideae; Nostocales;f__Nostocaceae;g__	VG	0.000031
Archaea; Euryarchaeota; Methanobacteria; Methanobacteriales;f__Methanobacteriaceae;g__Methanobrevibacter	BA	0.000031
Bacteria; Proteobacteria; Betaproteobacteria; Burkholderiales;f__Comamonadaceae;g__Limnobacter	MU	0.000028
Bacteria; Bacteroidetes; Bacteroidia; Bacteroidales;f__Bacteroidaceae;g__	TE	0.000024
Bacteria; Proteobacteria; Deltaproteobacteria; Myxococcales;f__OM27;g__	XP	0.000023
Bacteria; Firmicutes; Clostridia; Clostridiales;f__Tissierellaceae;g__Parvimonas	JL	0.000023
Bacteria; Actinobacteria; Acidimicrobiia; Acidimicrobiales;f__AKIW874;g__	QA	0.000023
Bacteria; Thermi; Deinococci; Deinococcales;f__Trueperaceae;g__Truepera	NS	0.000022
Bacteria; Cyanobacteria; Nostocophycideae; Nostocales;f__Nostocaceae;g__Nostoc	WG	0.000022
Bacteria; Firmicutes; Clostridia; Clostridiales;f__Gracilibacteraceae;Other	KJ	0.000022
Bacteria; Proteobacteria; Gammaproteobacteria; Xanthomonadales;f__Sinobacteraceae;g__	VQ	0.000021

Table S3: Blood analytes for all horses

Horse ID	Insulin (uIU/ml)	ACTH (pg/ml)	Cortisol (ug/dL)	Leptin (ng/ml)	Glucose (mg/dL)	Triglycerides (mg/dL)
2	19.70	35.8	7.4	9.54	88	54
3	17.14	11.7	4.44	10.02	85	29
4	14.25	23.9	4.81	11.22	86	38
5	11.00	26.3	3.56	4.15	81	15
6	7.99	63.8	7.97	8.04	94	25
7	15.90	20.4	5.16	ND	91	26
8	7.19	14.8	6.25	16.88	91	27
9	12.60	31.9	4.33	21.04	93	51
10	1.43	26.1	3.03	4.63	87	20
11	9.15	24.1	3.87	13.25	82	30
12	13.23	29.4	3.87	20.96	87	36
13	2.48	38.3	4.19	3.54	80	29

14	6.28	21.3	3.06	20.24	95	33
15	8.33	24.8	4.1	41.69	107	37
16	4.97	9.03	3.51	7.67	95	48
17	6.95	18.6	3.14	4.64	82	24
18	9.21	39	5.48	16.89	98	41
19	6.13	9.38	4.3	24.04	92	37
20	10.74	26.9	5.65	15.37	97	32
21	9.82	5.46	3.82	14.1	96	74
22	2.00	36.4	5.27	4.35	92	25
23	6.00	21.5	3.95	6.11	92	29
24	8.27	12.2	3.2	3.36	94	28
25	1.48	5.48	3.84	10	89	32
26	5.91	16.3	3.14	9.73	87	53
27	39.00	15.4	5.55	13.2	126	73
28	10.15	21.3	3.41	13.77	94	45
29	1.47	20.2	3.63	4.07	90	28
30	16.42	12.3	2.64	4.22	102	38
31	11.48	20.1	3.9	7.3	92	26
32	16.12	28.5	4.23	5.5	93	34
33	7.85	22.7	3.88	9.39	97	28
35	41.54	41.4	5.07	< 1.00	95	45
36	13.80	26.9	5.17	7.13	94	42
37	14.86	24.4	7.13	3.01	107	38
39	4.56	22.6	4.43	5.46	84	22
40	79.71	26.4	2.45	10.83	108	37
41	7.82	22.2	5.46	8.42	86	37
42	51.88	34.6	5.11	13.5	106	37
43	7.81	29.6	5.68	10.56	88	30
44	2.74	19	4.25	5.25	89	21
46	33.72	31.3	4.17	16.13	95	32
47	12.74	24.1	5.65	5.64	96	20
48	1.48	35.7	4.84	3.75	91	12
49	3.49	18.1	3.54	< 1.00	86	23
50	10.75	24.9	7.16	7.78	96	12
51	2.81	30.4	4.24	5.96	86	20
52	0.14	44.2	3.84	2.47	85	36
53	1.78	18.1	3.87	3.06	86	27
54	1.34	16.9	2.45	5.62	84	30
56	9.82	21.2	2.91	ND	107	ND
57	3.70	19.2	4.65	4.61	90	29
58	21.00	26.9	3.25	8.04	102	31

59	25.45	26.5	4.5	12.22	110	24
62	26.91	14.5	2.86	3.97	76	43
64	24.22	16.2	2.84	5.42	77	25
67	15.72	11.6	1.96	5.3	82	12
69	15.84	13.7	4.12	3.72	80	19
70	15.54	12.2	2.45	3.34	78	14
71	23.49	14.8	2.11	3.35	75	20
74	2.89	23	7.07	3.9	91	26
77	14.90	18.7	7.18	3.2	95	19
78	4.08	19.8	6.13	9.66	90	51
80	12.48	20.4	3.18	4.52	90	15
82	11.68	16.9	3.36	4.4	78	19
83	22.65	24.6	2.63	2.22	85	31
85	4.45	16.5	5.74	3.41	92	25
86	27.87	16.7	1.75	2.94	86	26
87	20.97	13.7	1.82	6.3	91	8
88	18.31	12.4	2.08	2.07	84	22
90	17.15	22.4	1.65	14.09	74	38
91	49.22	25.1	1.92	23.58	85	57
94	12.65	11.2	1.18	2.21	80	21
96	19.14	22.1	5.26	10.04	95	26
98	9.27	34.6	5.69	8.84	92	37
99	6.11	21.9	3.55	ND	95	28
108	18.85	15.9	1.74	18.61	89	40
119	31.32	19.3	4.91	9.67	95	15
