# Supplementary figures and images for: Microbiome and Blood Analyte Differences Point to Community and Metabolic Signatures in Lean and Obese Horses
Source: Front Vet Sci. 2018 Sep 20;5:225. doi: 10.3389/fvets.2018.00225 (PMC6158370; doi:10.3389/fvets.2018.00225)

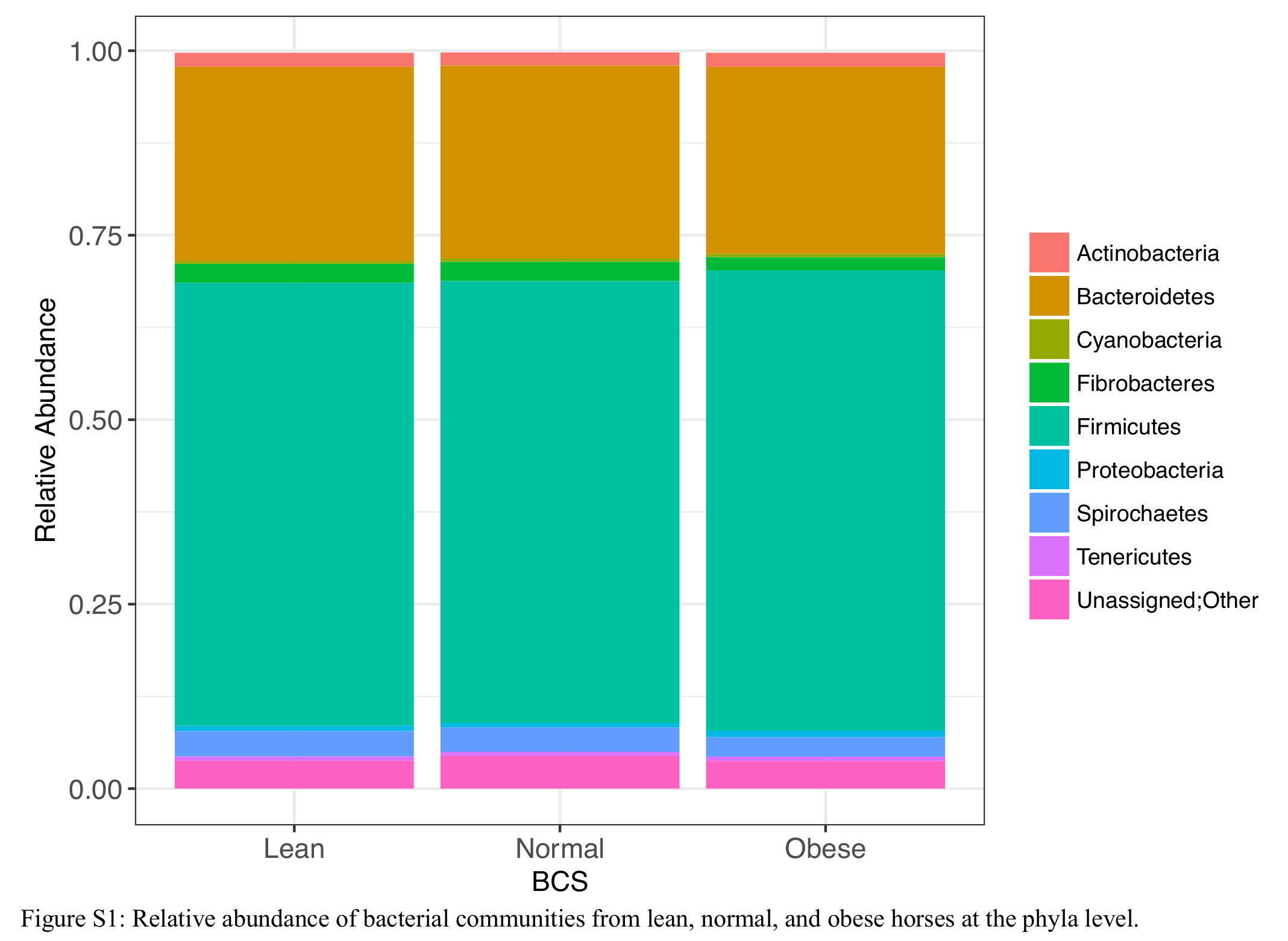

Supplement: Supplementary file 2 [file Image_1.tif]

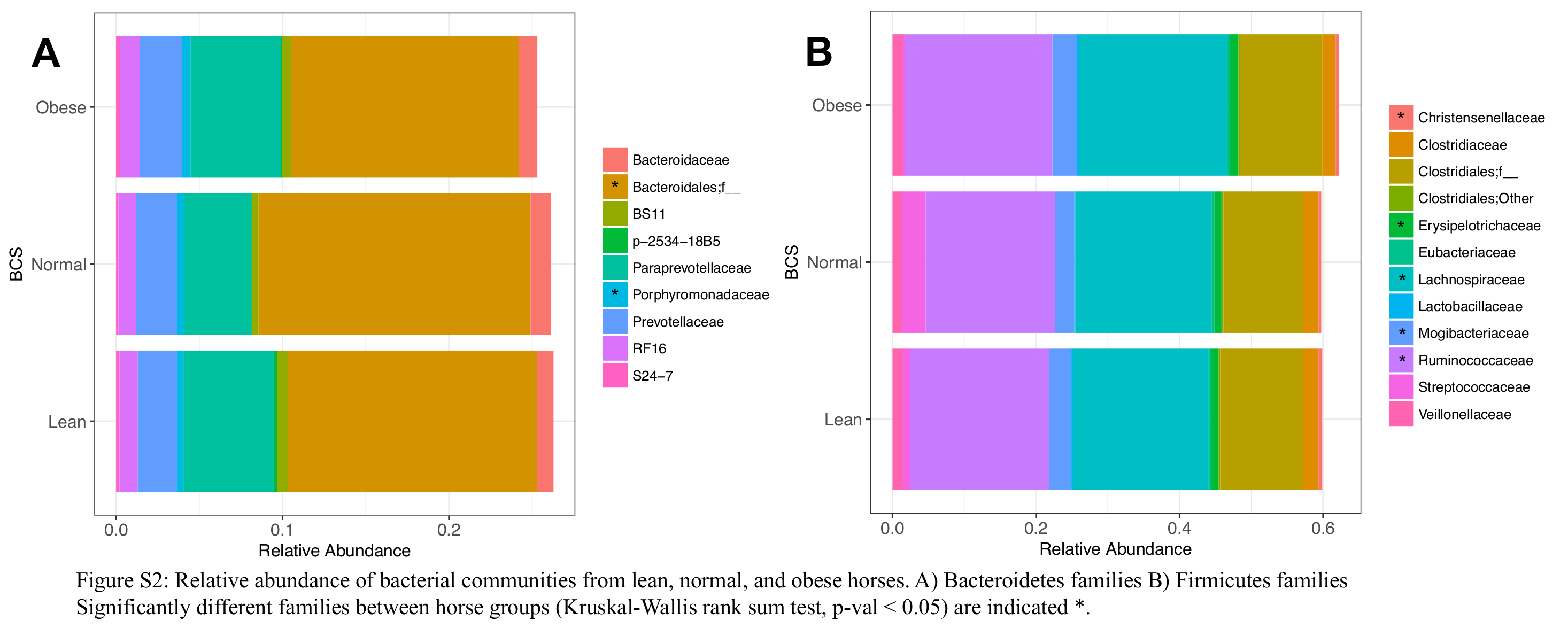

Supplement: Supplementary file 3 [file Image_2.tif]
